# Uncovering key salt-tolerant regulators through a combined eQTL and GWAS analysis using the super pan-genome in rice

**DOI:** 10.1093/nsr/nwae043

**Published:** 2024-02-05

**Authors:** Hua Wei, Xianmeng Wang, Zhipeng Zhang, Longbo Yang, Qianqian Zhang, Yilin Li, Huiying He, Dandan Chen, Bin Zhang, Chongke Zheng, Yue Leng, Xinglan Cao, Yan Cui, Chuanlin Shi, Yifan Liu, Yang Lv, Jie Ma, Wenchuang He, Xiangpei Liu, Qiang Xu, Qiaoling Yuan, Xiaoman Yu, Tianyi Wang, Hongge Qian, Xiaoxia Li, Bintao Zhang, Hong Zhang, Wu Chen, Mingliang Guo, Xiaofan Dai, Yuexing Wang, Xiaoming Zheng, Longbiao Guo, Xianzhi Xie, Qian Qian, Lianguang Shang

**Affiliations:** Shenzhen Branch, Guangdong Laboratory of Lingnan Modern Agriculture, Genome Analysis Laboratory of the Ministry of Agriculture and Rural Affairs, Agricultural Genomics Institute at Shenzhen, Chinese Academy of Agricultural Sciences, Shenzhen 518120, China; Shenzhen Branch, Guangdong Laboratory of Lingnan Modern Agriculture, Genome Analysis Laboratory of the Ministry of Agriculture and Rural Affairs, Agricultural Genomics Institute at Shenzhen, Chinese Academy of Agricultural Sciences, Shenzhen 518120, China; Shenzhen Branch, Guangdong Laboratory of Lingnan Modern Agriculture, Genome Analysis Laboratory of the Ministry of Agriculture and Rural Affairs, Agricultural Genomics Institute at Shenzhen, Chinese Academy of Agricultural Sciences, Shenzhen 518120, China; Shenzhen Branch, Guangdong Laboratory of Lingnan Modern Agriculture, Genome Analysis Laboratory of the Ministry of Agriculture and Rural Affairs, Agricultural Genomics Institute at Shenzhen, Chinese Academy of Agricultural Sciences, Shenzhen 518120, China; Shenzhen Branch, Guangdong Laboratory of Lingnan Modern Agriculture, Genome Analysis Laboratory of the Ministry of Agriculture and Rural Affairs, Agricultural Genomics Institute at Shenzhen, Chinese Academy of Agricultural Sciences, Shenzhen 518120, China; Shenzhen Branch, Guangdong Laboratory of Lingnan Modern Agriculture, Genome Analysis Laboratory of the Ministry of Agriculture and Rural Affairs, Agricultural Genomics Institute at Shenzhen, Chinese Academy of Agricultural Sciences, Shenzhen 518120, China; Shenzhen Branch, Guangdong Laboratory of Lingnan Modern Agriculture, Genome Analysis Laboratory of the Ministry of Agriculture and Rural Affairs, Agricultural Genomics Institute at Shenzhen, Chinese Academy of Agricultural Sciences, Shenzhen 518120, China; Shenzhen Branch, Guangdong Laboratory of Lingnan Modern Agriculture, Genome Analysis Laboratory of the Ministry of Agriculture and Rural Affairs, Agricultural Genomics Institute at Shenzhen, Chinese Academy of Agricultural Sciences, Shenzhen 518120, China; Shenzhen Branch, Guangdong Laboratory of Lingnan Modern Agriculture, Genome Analysis Laboratory of the Ministry of Agriculture and Rural Affairs, Agricultural Genomics Institute at Shenzhen, Chinese Academy of Agricultural Sciences, Shenzhen 518120, China; Institute of Wetland Agriculture and Ecology, Shandong Academy of Agricultural Sciences, Jinan 250100, China; Shenzhen Branch, Guangdong Laboratory of Lingnan Modern Agriculture, Genome Analysis Laboratory of the Ministry of Agriculture and Rural Affairs, Agricultural Genomics Institute at Shenzhen, Chinese Academy of Agricultural Sciences, Shenzhen 518120, China; Shenzhen Branch, Guangdong Laboratory of Lingnan Modern Agriculture, Genome Analysis Laboratory of the Ministry of Agriculture and Rural Affairs, Agricultural Genomics Institute at Shenzhen, Chinese Academy of Agricultural Sciences, Shenzhen 518120, China; Shenzhen Branch, Guangdong Laboratory of Lingnan Modern Agriculture, Genome Analysis Laboratory of the Ministry of Agriculture and Rural Affairs, Agricultural Genomics Institute at Shenzhen, Chinese Academy of Agricultural Sciences, Shenzhen 518120, China; Shenzhen Branch, Guangdong Laboratory of Lingnan Modern Agriculture, Genome Analysis Laboratory of the Ministry of Agriculture and Rural Affairs, Agricultural Genomics Institute at Shenzhen, Chinese Academy of Agricultural Sciences, Shenzhen 518120, China; Shenzhen Branch, Guangdong Laboratory of Lingnan Modern Agriculture, Genome Analysis Laboratory of the Ministry of Agriculture and Rural Affairs, Agricultural Genomics Institute at Shenzhen, Chinese Academy of Agricultural Sciences, Shenzhen 518120, China; Shenzhen Branch, Guangdong Laboratory of Lingnan Modern Agriculture, Genome Analysis Laboratory of the Ministry of Agriculture and Rural Affairs, Agricultural Genomics Institute at Shenzhen, Chinese Academy of Agricultural Sciences, Shenzhen 518120, China; State Key Laboratory of Rice Biology, China National Rice Research Institute, Hangzhou 310006, China; Shenzhen Branch, Guangdong Laboratory of Lingnan Modern Agriculture, Genome Analysis Laboratory of the Ministry of Agriculture and Rural Affairs, Agricultural Genomics Institute at Shenzhen, Chinese Academy of Agricultural Sciences, Shenzhen 518120, China; State Key Laboratory of Rice Biology, China National Rice Research Institute, Hangzhou 310006, China; Shenzhen Branch, Guangdong Laboratory of Lingnan Modern Agriculture, Genome Analysis Laboratory of the Ministry of Agriculture and Rural Affairs, Agricultural Genomics Institute at Shenzhen, Chinese Academy of Agricultural Sciences, Shenzhen 518120, China; Shenzhen Branch, Guangdong Laboratory of Lingnan Modern Agriculture, Genome Analysis Laboratory of the Ministry of Agriculture and Rural Affairs, Agricultural Genomics Institute at Shenzhen, Chinese Academy of Agricultural Sciences, Shenzhen 518120, China; Shenzhen Branch, Guangdong Laboratory of Lingnan Modern Agriculture, Genome Analysis Laboratory of the Ministry of Agriculture and Rural Affairs, Agricultural Genomics Institute at Shenzhen, Chinese Academy of Agricultural Sciences, Shenzhen 518120, China; Shenzhen Branch, Guangdong Laboratory of Lingnan Modern Agriculture, Genome Analysis Laboratory of the Ministry of Agriculture and Rural Affairs, Agricultural Genomics Institute at Shenzhen, Chinese Academy of Agricultural Sciences, Shenzhen 518120, China; Shenzhen Branch, Guangdong Laboratory of Lingnan Modern Agriculture, Genome Analysis Laboratory of the Ministry of Agriculture and Rural Affairs, Agricultural Genomics Institute at Shenzhen, Chinese Academy of Agricultural Sciences, Shenzhen 518120, China; Shenzhen Branch, Guangdong Laboratory of Lingnan Modern Agriculture, Genome Analysis Laboratory of the Ministry of Agriculture and Rural Affairs, Agricultural Genomics Institute at Shenzhen, Chinese Academy of Agricultural Sciences, Shenzhen 518120, China; Shenzhen Branch, Guangdong Laboratory of Lingnan Modern Agriculture, Genome Analysis Laboratory of the Ministry of Agriculture and Rural Affairs, Agricultural Genomics Institute at Shenzhen, Chinese Academy of Agricultural Sciences, Shenzhen 518120, China; Shenzhen Branch, Guangdong Laboratory of Lingnan Modern Agriculture, Genome Analysis Laboratory of the Ministry of Agriculture and Rural Affairs, Agricultural Genomics Institute at Shenzhen, Chinese Academy of Agricultural Sciences, Shenzhen 518120, China; Shenzhen Branch, Guangdong Laboratory of Lingnan Modern Agriculture, Genome Analysis Laboratory of the Ministry of Agriculture and Rural Affairs, Agricultural Genomics Institute at Shenzhen, Chinese Academy of Agricultural Sciences, Shenzhen 518120, China; Shenzhen Branch, Guangdong Laboratory of Lingnan Modern Agriculture, Genome Analysis Laboratory of the Ministry of Agriculture and Rural Affairs, Agricultural Genomics Institute at Shenzhen, Chinese Academy of Agricultural Sciences, Shenzhen 518120, China; Shenzhen Branch, Guangdong Laboratory of Lingnan Modern Agriculture, Genome Analysis Laboratory of the Ministry of Agriculture and Rural Affairs, Agricultural Genomics Institute at Shenzhen, Chinese Academy of Agricultural Sciences, Shenzhen 518120, China; Shenzhen Branch, Guangdong Laboratory of Lingnan Modern Agriculture, Genome Analysis Laboratory of the Ministry of Agriculture and Rural Affairs, Agricultural Genomics Institute at Shenzhen, Chinese Academy of Agricultural Sciences, Shenzhen 518120, China; Shenzhen Branch, Guangdong Laboratory of Lingnan Modern Agriculture, Genome Analysis Laboratory of the Ministry of Agriculture and Rural Affairs, Agricultural Genomics Institute at Shenzhen, Chinese Academy of Agricultural Sciences, Shenzhen 518120, China; State Key Laboratory of Rice Biology, China National Rice Research Institute, Hangzhou 310006, China; National Key Facility for Crop Gene Resources and Genetic Improvement, Institute of Crop Science, Chinese Academy of Agricultural Sciences, Beijing 100081, China; State Key Laboratory of Rice Biology, China National Rice Research Institute, Hangzhou 310006, China; Institute of Wetland Agriculture and Ecology, Shandong Academy of Agricultural Sciences, Jinan 250100, China; Shenzhen Branch, Guangdong Laboratory of Lingnan Modern Agriculture, Genome Analysis Laboratory of the Ministry of Agriculture and Rural Affairs, Agricultural Genomics Institute at Shenzhen, Chinese Academy of Agricultural Sciences, Shenzhen 518120, China; State Key Laboratory of Rice Biology, China National Rice Research Institute, Hangzhou 310006, China; Yazhouwan National Laboratory, Sanya 572024, China; Shenzhen Branch, Guangdong Laboratory of Lingnan Modern Agriculture, Genome Analysis Laboratory of the Ministry of Agriculture and Rural Affairs, Agricultural Genomics Institute at Shenzhen, Chinese Academy of Agricultural Sciences, Shenzhen 518120, China; Yazhouwan National Laboratory, Sanya 572024, China

**Keywords:** super pan-genome, expression quantitative trait loci, GWAS, salt tolerance, rice

## Abstract

For sessile plants, gene expression plays a pivotal role in responding to salinity stress by activating or suppressing specific genes. However, our knowledge of genetic variations governing gene expression in response to salt stress remains limited in natural germplasm. Through transcriptome analysis of the Global Mini-Core Rice Collection consisting of a panel of 202 accessions, we identified 22 345 and 27 610 expression quantitative trait loci associated with the expression of 7787 and 9361 eGenes under normal and salt-stress conditions, respectively, leveraging the super pan-genome map. Notably, combined with genome-wide association studies, we swiftly pinpointed the potential candidate gene *STG5*—a major salt-tolerant locus known as *qSTS5*. Intriguingly, STG5 is required for maintaining Na^+^/K^+^ homeostasis by directly regulating the transcription of multiple members of the *OsHKT* gene family. Our study sheds light on how genetic variants influence the dynamic changes in gene expression responding to salinity stress and provides a valuable resource for the mining of salt-tolerant genes in the future.

## INTRODUCTION

More than 1 billion hectares of land are significantly impacted by salinity, according to the Food and Agriculture Organization (FAO, 2015). Due to limitations in available fresh water, soil salinity has developed into a substantial environmental concern for crop growth and production globally. Moreover, given the negative consequences of climate change and increasing populations, there is an urgent requirement for breeding high-yielding and stress-tolerant varieties for the utilization of saline land for crop production.

Rice (*Oryza sativa* L.) is grown across the globe and one of the most important staple food crops. However, it is the most sensitive crop to salinity stress among the cereals, with 30 mM of NaCl strongly reducing growth and resulting in yield loss [1]. A 50% yield reduction was observed in soil with 60 mM of NaCl in many high-yielding varieties [2]. The adverse effects on plants resulting from salinity stress are primarily manifested in three ways: osmotic stress, ionic stress and oxidative stress. Osmotic stress predominantly limits water uptake, while excessive accumulation of toxic Na^+^ and Cl^−^ results in ionic imbalances, and oxidative damage caused by excessive accumulation of reactive oxygen species (ROS) leads to the hampering of plant growth and senescence [3,4]. Plant response to salt stress is complex and controlled by multiple genes at transcriptional or post-transcriptional levels.

Salinity tolerance is a polygenic trait governed by quantitative trait loci (QTL) [5]. To date, while several QTL related to salt tolerance have been identified, only a small number of major salt-tolerance genes have been uncovered, including *SKC1* (*shoot K^+^ content 1*), *HST1* (*hitomebore salt tolerant 1*), *STH1* (*salt tolerance and heading date 1*) and *RST1* (*rice salt tolerant 1*) [6–9]. Among these genes, *SKC1*, which encodes a member of the HKT-type (high-affinity K^+^ transporter) Na^+^ transporter *OsHKT1;5* (*high-affinity K^+^ transporter 1;5*), is one of the earliest cloned salt-associated genes and widely applicable for salt-tolerant breeding. Recent studies have identified two salt-tolerance-related genes (*OsWRKY53* and *OsMKK10.2* (*mitogen activated protein kinase 10.2*)) through genome-wide association studies (GWAS), responsible for the repressed transcription of *OsHKT1;5* in promoting ion homeostasis [10]. Nonetheless, the number of known salt-tolerant genes is still limited and our understanding of them is not as complete as it could be. This issue is present not only in rice, but also in other major glycophyte cereal crops such as maize and wheat. Dozens of QTL related to maize salt tolerance have been identified [11], while only some of these QTL (*ZmNAC1* (*Na^+^ CONTENT 1*)/*ZmHKT1* and *ZmNC2*/*ZmHAK4* (*high-affinity K^+^ transporters 4*)) have been genetically validated [12,13]. Due to the genome complexity, the wheat salt-tolerance QTL and genes have also been relatively less identified. *Kna1* (*TaHKT1;5-D*), *Nax1* (*Na^+^ exclusion 1*/*TmHKT7-A2*) and *Nax2* (*Na^+^ exclusion 2*/*TmHKT1;5-A*) are important HKT-type transporters conferring salt tolerance in wheat [14]. Further research is necessary to unearth major salt-tolerance genes and investigate their regulatory networks in rice, which can provide a reference for understanding the salt tolerance of maize, wheat and other crops.

Gene expression is essential for linking genotypes to the phenotypes of an organism, which is of critical importance to stress response and adaptation. The genetic basis of gene expression variability, also known as expression quantitative trait loci (eQTL), was assessed across animals and plants. In previous studies, the eQTL analysis has used inbred lines, such as barley, maize and *Arabidopsis* [15]. Using natural populations for eQTL analysis can provide novel and significant insights into the genetic basis of natural variants at the expression level and phenotypic variants, which has been conducted in lettuce [16], cotton [17], rice [18] and humans [19]. For instance, across leaf transcriptomes from rice accessions, the transcription factor of *bHLH026* was identified at a distant eQTL hotspot, responsible for regulating diterpenoid antitoxin synthesis and enhancing disease resistance [18]. However, because of the spatio-temporal reliance on gene expression, the eQTL architecture dynamically changes throughout development or in response to diverse stress. Comparisons of these dynamic changes allow us to more adequately understand the regulatory mechanisms of gene expression and give us important information for future research. In maize, transcriptomes under various conditions were analysed, reflecting a comprehensive and dynamic genetic architecture of gene expression responding to drought stress [20]. According to this achievement, the researchers successfully determined the molecular basis of significant drought-resistant maize germplasm [21]. Previously, the transcriptome profiles in response to salt stress were mainly analysed between salt-tolerant and salt-sensitive rice varieties [22,23]. eQTL determination at the natural population level and in dynamic environments aids in a comprehensive and precise understanding of gene expression architecture plasticity in dynamic conditions. However, genome-wide comparisons of eQTL under normal and salt stress have not often been conducted in major crops, especially in rice.

The super pan-genome of rice and complete sequences of *Nipponbare* (T2T-NIP) provide a more comprehensive genomic perspective, with more abundant information and comprehensive genetic diversity throughout evolutionary processes [24,25]. The rice Mini-Core Collection generated from global data, as well as their representativeness of genetic and phenotypic diversity in global rice germplasms, were employed in the study of gene expression variation and associated regulatory genomic elements in diverse environments. This is more advantageous for investigating the regulatory network responsible for salt-stress response and for salt-tolerant breeding.

In this study, we compared the analysis of transcriptomic data from across 202 accessions obtained from the rice Mini-Core Collection under normal and salt-stress conditions. From these accessions, we obtained 12 898 differentially expressed genes (DEGs) in the population level. Based on the super pan-genome map, 22 345 and 27 610 eQTL associated with the expression of 7787 and 9361 eGenes were respectively identified under normal and salt-stress conditions, giving us abundant resources for identifying salt-associated genes. Additionally, we conducted GWAS using pan-genomic variations and eight salt-tolerance traits to identify 28 salt-tolerance-associated loci. Among these loci, *qSTS5* (*salt tolerance site 5*) was consistently detected across multiple phenotypes. Combining DEGs, eQTL and GWAS analysis, we determined that *STG5* (*LOC_Os05g49700, salt tolerance gene 5*) was the most important causal candidate gene of *qSTS5* and further clarified that *STG5* conferred salt tolerance through the regulation of Na^+^ and K^+^ homeostasis.

## RESULTS

### Transcriptome analysis of variable gene expression under normal and salt-stress conditions

**Figure 1.**
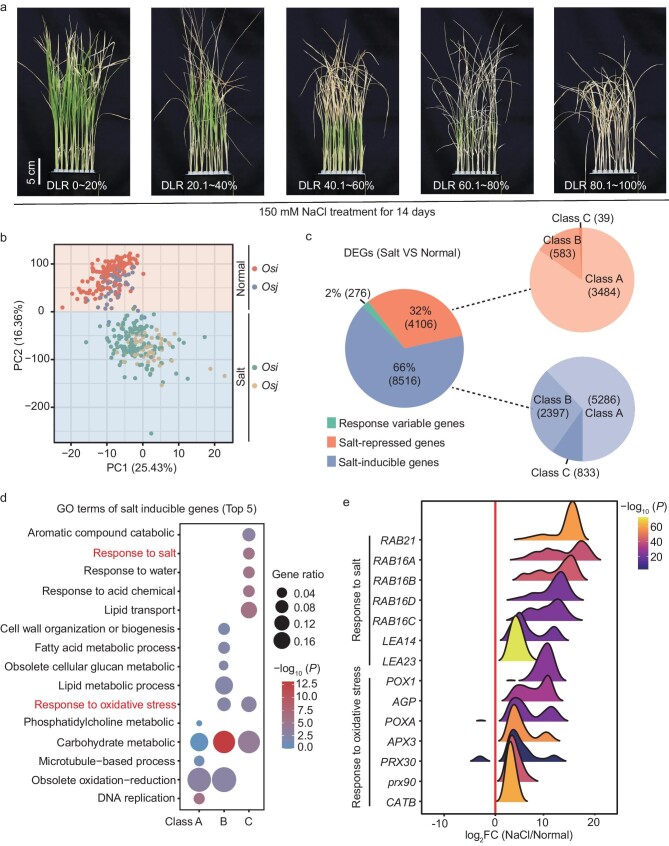
Transcriptome analysis of gene expression profiling across 202 Mini-Core rice accessions compared normal with NaCl-stress conditions. (a) Salt-tolerance level of representative rice varieties of this panel after 150 mM of NaCl treatment for 2 weeks. DLR, dead leaf rate. (b) The PCA analysis separated expression of identical genotypes under normal and salt-stress conditions using read count of expression genes. (c) Percentage of three clusters of DEGs (class A, 20 < *n* ≤ 101, 10%–50%; class B, 101 < *n* ≤ 182, 50%–90%; class C, 182 < *n* ≤ 202, 90%–100%); *n* stands for the proportion of samples. (d) Top five GO terms of salt-inducible genes cluster. (e) Distribution of log_2_ FoldChange of NaCl/normal for response to oxidative stress and response to salt terms of (d).

Prior studies have provided a graph-based super pan-genome of rice comprising a 251-accession panel from around the world [24]. A subpanel of 202 diverse rice genotypes, including *indica* (139) and *japonica* (63), was chosen to characterize how genetic variants impact gene expression when responding to salinity stress. The principal component analysis (PCA) also indicated the presence of a clear subpopulation structure (Fig. S1a). Initially, we characterized the salt-tolerance traits of this subpanel. Seedlings (16 days after sowing) for each of the accessions were subjected to 150 mM of NaCl stress for 14 days. Following this exposure, the symptomatic presentation on plant leaves and roots was observed and recorded based on eight criteria (root length, shoot fresh weight, shoot dry weight, root fresh weight, root dry weight, survival rate (SR), dead leaf rate (DLR) and salt-tolerance level (STL)). The STL was determined using the percentage of dead leaf blade tissue [26]. These salt-tolerance-linked traits were examined using at least two independent experiments. Phenotypic characteristics of eight salt-related traits under normal and 150 mM of NaCl conditions among 202 rice accessions are displayed in Table S1. Generally, this population had diverse salt tolerance among varieties, with some accessions exhibiting more tolerance than other well-known varieties (Fig. 1a and Fig. S1b–e).

Seedlings were exposed to 24 hours of salinity stress (150 mM of NaCl) conditions and a group of two or three individuals for each genotype combination was employed for RNA-seq. The RNA-seq data were aligned to the rice *Nipponbare* reference genome (MSU 7.0) for quantification and all samples exhibited high alignment rates, with an average rate of unique mapped reads of 82.0%. After the removal of genes with no read counts across 90% of samples, we obtained a gene count matrix containing reads assigned to a set of 36 025 genes. Initially, PCA was conducted to assess the overall variability in the transcriptomes of all samples. The results indicated that the first two principal components distinguished the samples according to salt treatment, demonstrating the significant impact of salinity stress on gene expression patterns (Fig. 1b).

We established the cut-off for each gene, with the value of log2FoldChange ≥ 2 relative to the normal condition in samples and detected in >10% of the accessions as the cluster of DEGs. Based on these criteria, a total of 12 898 DEGs were uncovered. Next, we separated these DEGs into salt-inducible genes and salt-repressed genes (genes were upregulated or downregulated across >20 varieties), accounting for 66% (8516) and 32% (4106), respectively. In addition to up- and downregulated gene clusters, we characterized these 276 genes (2%) as responsive-variable genes that existed as both up- and downregulated clusters and response diversities in these rice accessions (Fig. 1c). Based on the percentages of samples, we classified salt-inducible and salt-repressed genes into three clusters (class A, 20 < *n* ≤ 101, 10%–50%; class B, 101 < *n* ≤ 182, 50%–90%; class C, 182 < *n* ≤ 202, 90%–100%) (Fig. 1c). The heat map of three cluster genes, including salt-inducible, salt-repressed and responsive-variable genes, exhibited variable salt-responsive patterns throughout the rice panel, demonstrating the key response diversities in different genetic backgrounds. Moreover, varieties from the same origin (for example *Os. indica* and *Os. japonica*) did not cluster together based on gene expression patterns, indicating that variations in gene expression were not entirely dependent on genomic similarity and were also dependent on the environment (Fig. S2a–c). Salt stress results in the cellular accumulation of ROS and substantially damages cellular structures and macromolecules, including DNA, lipids and enzymes. The top five Gene Ontology (GO) terms pertaining to salt-inducible genes suggested that class A genes were related to DNA replication (GO: 0006260), obsolete oxidation–reduction process (GO: 0055114), microtubule-based process (GO: 0007017), as well as carbohydrate and phosphatidylcholine metabolic process (GO: 0005975, GO: 0046470) (Fig. 1d). In addition to obsolete oxidation–reduction and carbohydrate metabolic processes, the processes of response to oxidative stress and lipid metabolic were also significantly abundant in cluster B. The class C genes were primarily enriched in lipid transport and localization, as well as response to acid, chemical, water and salt-stress categories, determining the ability of salt tolerance (Fig. 1d). Notably, we identified that many *Prxs* linked to oxidative stress and *LEAs* (late embryogenesis-abundant) were associated with abiotic stress tolerance and were consistently upregulated in response to salt stress in the vast majority of rice accessions (Fig. 1e). Moreover, salt stress resulted in rapid stomatal closure, reducing the plant's ability to assimilate CO_2_ and limiting photosynthesis. A GO enrichment analysis of a cluster of salt-repressed genes revealed several significant GO enrichments associated with photosynthetic processes (i.e. GO: 0015979) (Fig. S3a). Consistently, the 15 members enriched in photosynthesis were downregulated compared with normal conditions (Fig. S3b). The cluster of 276 genes was particularly enriched in the GO terms related to oxidation and reduction, which are closely related to salt tolerance in the top five terms (Fig. S3c). Among these, 29 members displayed a responsive-variable pattern across 200 genotypes (Fig. S3d), indicating that natural variability played a clear regulatory role in gene expression across various rice accessions.

Prior studies have uncovered that 157 genes are critical for salinity stress response [27]. We noted that 90 out of 157 genes exhibit significant enrichment in the DEGs data set compared with all genes of the entire genome (*P* = 6.620e^−21^), demonstrating that the DEGs data set is very useful for identifying novel salt-tolerance regulators. We are interested in gene expression variations in response to stress across the different accessions. Clustering of gene expression patterns suggested that 64 were generally upregulated throughout many accessions, including genes for Na^+^ and K^+^ transporter (*OsHKT2;1, OsHKT1;1, HAK21, OsHKT2;4, SKC1*), and antioxidative pathway (*SIK1* (*stress-induced protein kinase gene 1*) and *APXs* (*ASCORBATE PEROXIDASE*) (Fig. S4a). A total of 24 genes were mostly downregulated under salinity stress, such as *DST* (*Drought and Salt tolerance*) and *DCA1* (*DST Co-activator 1*), which function as negative regulators of salt tolerance. Intermediate filament-like protein was characterized as a *Saltol* QTL [28] to confer salt tolerance by maintaining a favorable K^+^/Na^+^ ratio (Fig. S4b). Additionally, *JAZ9* (*JASMONATE ZIM 9*) and *HAK1* exhibited diverse response directions among the 202 investigated accessions (Fig. S4c). The diversity in response intensity and direction demonstrated that natural variants underscore the expression of these genes and contribute to the variation in salinity response across these rice accessions.

### Genome-wide mapping of static and dynamic eQTL in response to salinity stress

**Figure 2.**
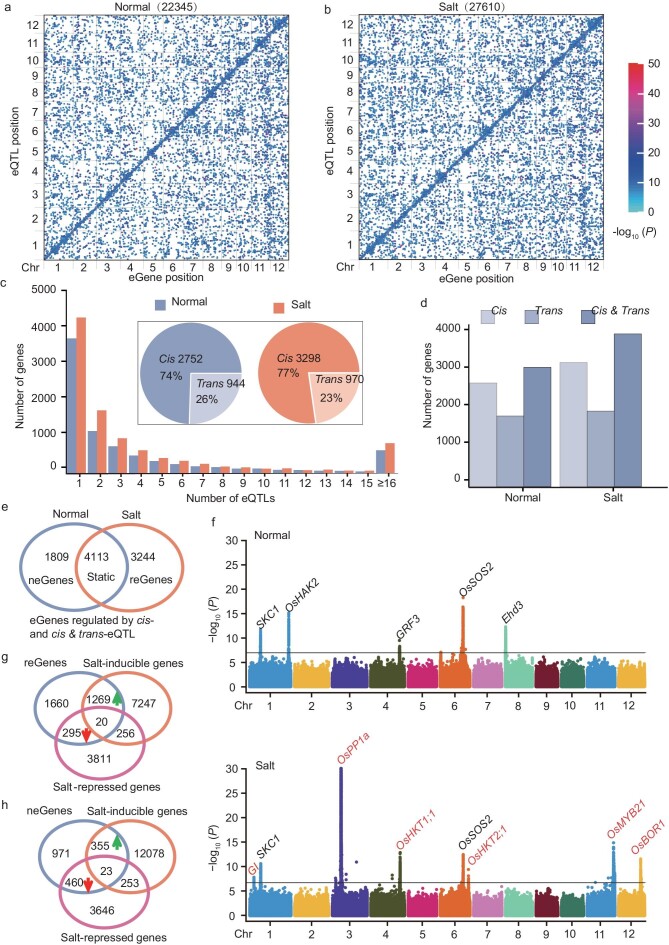
Large-scale *cis-* and *trans-*eQTL identified using transcriptome data under normal and NaCl-stress conditions. (a and b) Genome distribution of eQTL and their regulatory genes (eGenes) on 12 chromosomes under (a) normal and (b) salt conditions, respectively. The *x*-axis and *y*-axis respectively indicate the eGene and lead SNP position on each chromosome. Each dot indicates a *trans*-eQTL and the diagonal line shows a *cis-*eQTL. (c) Number of genes with one or more significant peaks and the percentage of *cis-* and *trans-*eQTL regulated genes with only one peak (pie plot) under normal and NaCl conditions. (d) Distribution of number of genes regulated by *cis-* or *trans*-eQTL and both *cis-* or *trans*-eQTL. (e) Venn diagram showing the static and dynamic eGene regulated by *cis-*eQTL and both *cis-* and *trans-*eQTL. (neGene and reGene represent that the eGene was only regulated by eQTL under normal and salinity stress conditions, respectively.) (f) Combined Manhattan plot showing the static, dynamic *cis-*eQTL detected for individual eGenes; e.g. *SKC1* and *OsSOS2* belonged to the static eGenes regulated by *cis-*eQTL; *OsHAK2, GRF3* and *Ehd3* only appeared under normal conditions that were contained in the neGenes cluster; *GI, OsPP1a, OsHKT1;1, OsHKT2;1, OsMYB21* and *OsBOR1* were considered as reGenes only detectable under salt-stress conditions. The black line indicates the genome-wide significant threshold (*P* = 1.82e^−7^). (g and h) Venn diagram showing reGenes (g), neGenes (h) and DEGs cluster.

To map regulatory variants that may impact gene expression levels or responses to salinity stress in rice germplasms, we retained genes with average FPKM (Fragments Per Kilobase Million) of >0.1, resulting in 23 736 and 26 450 genes expressed under normal and salt-stress conditions, respectively. Of these genes, 22 915 were commonly expressed under both conditions, while 821 and 3535 genes were uniquely detected and expressed under normal or salt-stress conditions only. We conducted eQTL mapping using normal and salinity stress expression data to map the genomic elements influencing transcript levels of expressed genes using a set of 5 491 340 SNPs (Single Nucleotide Polymorphisms) generated from the super pan-genome of rice. The identified eQTL were classified based on the most significant SNP. Associated SNPs located in the surrounding 200 Kb of the targeted genes were identified as *cis-*eQTL, while SNPs located >200 Kb from the target gene or on different chromosomes were classified as *trans*-eQTL. In plotting the start position of the lead SNP of the eQTL against the start positions of the eGene, a line along the diagonal was observed reflecting *cis* regulations. A total of 49 955 eQTL (encompassing 22 345 under normal conditions and 27 610 under salt stress) were detected, capturing an unprecedented range of regulatory variants governing gene expression in response to salt stress (Fig. 2a and b). As for regulatory variants, 5924 and 7358 *cis-*eQTL were identified under normal and salt-stress conditions, respectively, while 16 421 and 20 252 *trans*-eQTL were found under normal and salt stress, respectively (Fig. S5a). According to eQTL occurrence in different conditions, the eQTL for some genes consistently detected under normal and NaCl treatments were identified to be static eQTL, while those detected under either NaCl or normal conditions were designated as dynamic eQTL in response to salt stress. Therefore, 10 149 were recognized as static while 29 657 were recognized as dynamic eQTL (Fig. S5b, left). Among them, 4109 static *cis*-eQTL were stably identified under normal and salt-stress conditions. Importantly, across 5064 dynamic *cis*-eQTL, 3249 were only detected under salt-stress conditions (Fig. S5b, middle). Regarding the *trans*-eQTL, 5268 static and 26 137 dynamic *trans-*eQTL were identified, respectively (Fig. S5b, right).

With respect to eGenes (the gene regulated by eQTL were referred to as eGenes), we identified 7787 and 9361 eGenes with between 1 and 15 independent eQTL peaks under normal and salt-stress conditions, respectively (Fig. 2c). The small minority (618 under normal conditions and 813 under salt stress) for which >15 distinct peaks were identified were excluded from downstream analyses due to strong and scattered signals throughout the genome, which were inconsistent with the accurate identification of causal loci. Among 3696 (normal) and 4268 (salt-stress) eGenes with single discrete eQTL peaks, 2752 (75%) and 3298 (77%) contain *cis-*eQTL in normal and salt-stress environments, and the remaining are eGenes containing only *trans*-eQTL (encompassing 944 in normal conditions and 970 in salt-stress conditions) (Fig. 2c). In total, in terms of eGenes under normal conditions, 2753 and 1865 eGenes are regulated by only *cis-* and *trans-*eQTL, respectively, while 3169 eGenes are controlled by both *cis-* and *trans*-eQTL. Similarly, 3298, 2004 and 4059 eGenes are regulated by *cis*-eQTL or *trans*-eQTL or both *cis*- and *trans*-eQTL (Fig. 2d). These findings indicated that *cis-*eQTL have a greater effect on gene expression variations when comparing the explanation rate (*r*^2^) of SNPs in the *cis-* and *trans-*eQTL (Fig. S5c), consistently with previous studies in rice and maize [18,20]. Therefore, the eGenes regulated by *cis-*eQTL or regulated by both *cis-* and *trans*-eQTL were particularly interesting, as they reflected the variable level of gene expression under salt stress. Based on the occurrence of eGenes under different conditions, 4113 belonged to static eGenes, 1809 neGenes (these eGenes were only regulated by the eQTL under normal conditions that were defined as normal eGene, neGene) and 3244 reGenes (these eGenes were only regulated by the eQTL under salt-stress conditions that were defined as responsive eGene, reGene) pertained to dynamic eGenes under the normal and salt environments, respectively (Fig. 2e). Analogously, 2076 static, 2958 neGenes and 3987 reGenes were regulated by *trans*-eQTL (Fig. S5d). For instance, significant and unique association peaks were identified under two conditions for genes involved in salt response pathways (*SKC1* and *OsSOS2* (*Salt Overly Sensitive 2*)), which are supposed to be static eGenes. In addition to growth-regulating factor *GRF3* and heading date regulator *Ehd3*, the potassium transporter gene *OsHAK2* was detected only under normal conditions, pertaining to neGenes (Fig. 2f). Remarkably, among 5033 dynamic eGenes, 3244 were detected only under NaCl-stress treatment. Among them, the association signals of *GI* (*GIGANTEA*), *OsPP1a* (*Protein Phosphatase 1a*), *OsHKT1;1, OsHKT2;1, OsMYB21* and *OsBOR1* (*Boron 1*) were undetected under normal conditions, but highly increased upon stress treatment (Fig. 2f). These data suggest that local variants potentially confer stress responsiveness on these genes across diverse rice genotypes.

Through population transcriptome and eQTL analysis, we identified genetic data sets that may be associated with salinity stress response. To further investigate the biological significance of the DEGs and eGenes in regulating the salt-stress response, we found that reGenes exhibit significant enrichment (*P =* 6.85e^−98^) in the salt-inducible genes cluster through chi-square testing (Fig. S6a). It was noteworthy that 1269 of 3244 reGenes were enriched in the salt-inducible gene class, while only 295 were classified in salt-repressed genes (Fig. 2g), indicating that the reGenes were often linked to gene activation under salt stress. Additionally, 355 and 460 neGenes were respectively upregulated and downregulated (Fig. 2h). GO term analysis of four gene categories demonstrated that the salt-repressed gene cluster was involved in the metabolic process, while the salt-inducible genes played an important role in stress response (Fig. S6b and c). In particular, the class A cluster consisted of 1269 genes that were mainly responsive to oxidative stress (Fig. S6c). Additionally, 733 upregulated and 738 downregulated eGenes overlapped static eGenes, functioning in multiple metabolic processes (Fig. S6d and e). Overall, the eGene and DEGs data sets provide a solid foundation and a resource of salt-associated genes for investigating salt-tolerance-associated genes in the future.

### Explaining phenotypic changes through genomic and transcriptomic variations

**Figure 3.**
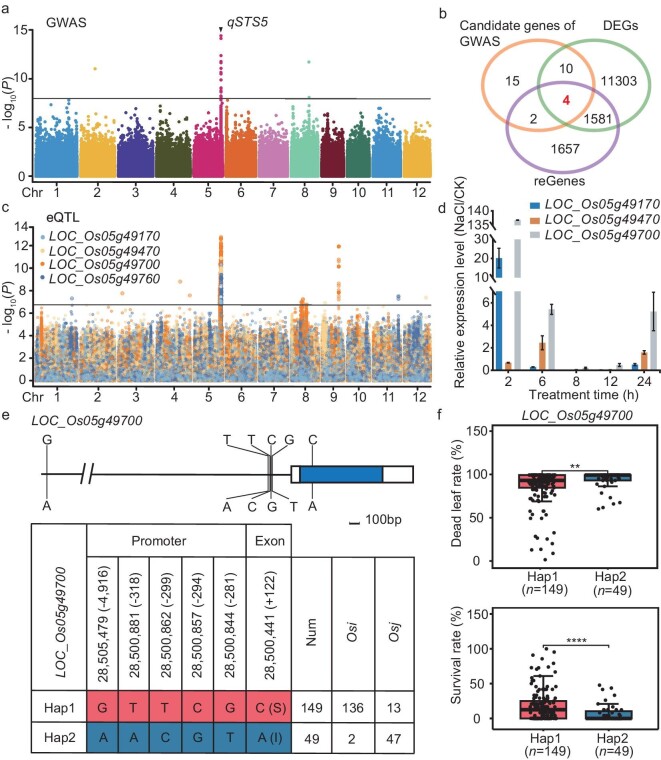
Combined GWAS with eQTL to identify the causal candidate gene of *LOC_Os05g49700*. (a) Manhattan plots for salt-tolerance level using a Genome-wide Efficient Mixed Model Association (GEMMA) in 202 *O. sativa* accessions. The black line indicates the genome-wide significant threshold (*P* = 1.08e^−8^), and the black triangle shows the peak on chromosome 5 and is named as *qSTS5* (*Salt Tolerance Site 5*). (b) Venn diagram showing the unigenes and the shared genes among candidate genes of GWAS, DEGs and reGenes. Different colors represent one gene set of unigenes and the values show the number of unigenes that were shared or unique among gene sets. The sum of all numbers inside the circle indicates the total number of unigenes in this set, and the intersection of circles shows the number of unigenes that overlapped among these gene sets. (c) Manhattan plots of eQTL for the four genes shown in (b) with significant and unique association peaks. (d) The relative expression levels of candidate genes under NaCl-stress to normal conditions. Rice *ACTIN* was used for the internal reference. Error bars represent the mean ± SE of three technical repeats; two biological replicates were conducted and obtained similar results. (e) The promoter and gene structure of *STG5* and DNA polymorphisms, and haplotypes (Hap) of *STG5* among these accessions based on the SNP of promoter and exon. (f) Analysis of dead leaf rate and survival rate in these accessions with the identical genotypes of *STG5*. The 149 accessions with *STG5^Hap2^* and 49 accessions with *STG5^Hap1^* are included in this analysis. Data represent means ± SD. *P*-values were determined using Student's *t*-test. **P < 0.01; ****P < 0.0001.

We investigated the salt-tolerance-related traits in the panel of diverse genotypes, which displayed a diversity of salinity tolerance among various rice varieties. To uncover factors responsible for regulating salt tolerance in rice, we conducted GWAS using the same SNP data set as the eQTL identification, based on the mixed linear model. Based on eight salt-tolerance traits, a total of 28 associated loci were uncovered, including similar loci with previous studies and new salt-stress-responsive loci (Table S2). Among them, a peak at 28 351 660 bp in chromosome (Chr.) 5 was identified as *qSTS5* in multiple phenotypes, including SR, DLR and STL, over two independent experiments. The strongest associated signals were detected alongside the ideal distribution of observed *P*-values based on QQ (quantile–quantile) plot analysis (Fig. 3a and Fig. S7a–d). To validate the reliability of *qSTS5*, we compared the salt tolerance of CSSLs (chromosome segment substitution lines) using *Nipponbare* as the recipient parent and *SR86* (*Sea rice 86*) and *PA64s* as donor parent, respectively. As a result, CSSLs (N-S-2/3/4^SR86^ and N-P-3^PA64s^) were more tolerant to salt stress compared with CSSLs (N-S-1^NIP^ and N-P-1/2^NIP^) (Fig. S8). It was assumed that *qSTS5* was a good candidate for rice salt tolerance and we therefore focused on it for further analysis. We then preferentially focused on 200 Kb upstream and downstream surrounding the target *qSTS5* peak region and identified 97 candidate genes within 546.49 Kb of the genomic region. Through further haplotype analysis based on non-synonymous mutations, the candidate genes were narrowed to 31 (Fig. S9). Remarkably, four members (*LOC_Os05g49170, LOC_Os05g49470, LOC_Os05g49700* and *LOC_Os05g49760*) of GWAS candidate genes in this region overlapped with DEGs and reGene data sets (Fig. 3b). Upon examination of eQTL results for these representatives, we determined that significant and unique association peaks were identified (Fig. 3c). Furthermore, we used the *coloc* R package to determine the co-localization between eQTL and GWAS surrounding these four genes, among which three genes (*LOC_Os05g49170, LOC_Os05g49470* and *LOC_Os05g49700*) were detected (COLOC.PP3 > 0.7) using the *coloc* R package (Table S3). Subsequently, quantitative PCR (qPCR) was conducted to compare the expression levels of three genes under normal and NaCl-stress conditions at various treatment time points. We noted that NaCl stress significantly induced their transcription, consistently with the transcriptome data set at the population level, showing that these three candidate genes were DEGs of salt-inducible gene clusters (Fig. 3d). Interestingly, among them, *LOC_Os05g49700* had the highest expression level following NaCl treatment (Fig. 3d). To further identify the most causal gene of *qSTS5*, we generated the null mutant of *LOC_Os05g49470* by employing CRISPR/Cas9 technology, while the null mutant of *LOC_Os05g49470* (*m470*) showed similar salinity sensitivity to wild-type control with 150 mM NaCl treatment after 12 days (Fig. S10a–e). Additionally, we identified the solitary homologous genes of *LOC_Os05g49170* in *Arabidopsis* (*AT1G29395, AtCOR314*) and subjected the mutant (*atcor314*) to salt-stress treatment (Fig. S10f–j). The results showed that the loss of function of this gene in *Arabidopsis* did not affect the salt tolerance, implying that *LOC_Os05g49170* may not regulate salt tolerance in rice. Taken together, the eQTL peak plot of *LOC_Os05g49700* was the most significant, which prompted us to focus further on *LOC_Os05g49700*, named *STG5* (*salt tolerance gene 5*).

Particularly, the *cis*-eQTL significant peak of *STG5* under NaCl-stress treatment overlapped with the same region as the GWAS results, while no signals appeared under normal conditions (Fig. S11a). Interestingly, we identified that the leading SNP of eQTL (chr5: 28505479) is within 5 Kb of the *STG5* locus and is completely linked to other SNP loci located in the promoter and exon of *STG5*, respectively (Fig. 3e and Fig. S11c). Based on sequence variations of these SNP sites across 198 rice accessions, after four outliers were removed, they were classified into two haplotypes (Hap1 and Hap2) of *STG5.* Additionally, we identified a non-synonymous mutation in the exon, with Ser mutated to Ile (Fig. 3e), which may result in the reduction of phosphorylation sites to affect the protein function. Furthermore, we observed that the rice accession containing Hap1 had a much higher SR and lower DLR than those with Hap2 under NaCl treatment (Fig. 3f), indicating that Hap1 confers stronger salt tolerance than Hap2. Additionally, we found that there was no obvious difference in the FPKM value between Hap1 and Hap2 under normal conditions, while the FPKM value was significantly higher in Hap2 under NaCl stress (Fig. S11b and d). Therefore, we predicted that this natural variation in *STG5* may have a genetic effect on salt tolerance.

### *STG5* functions as a key salt-tolerant regulator by maintaining Na^+^/K^+^ homeostasis in rice

**Figure 4.**
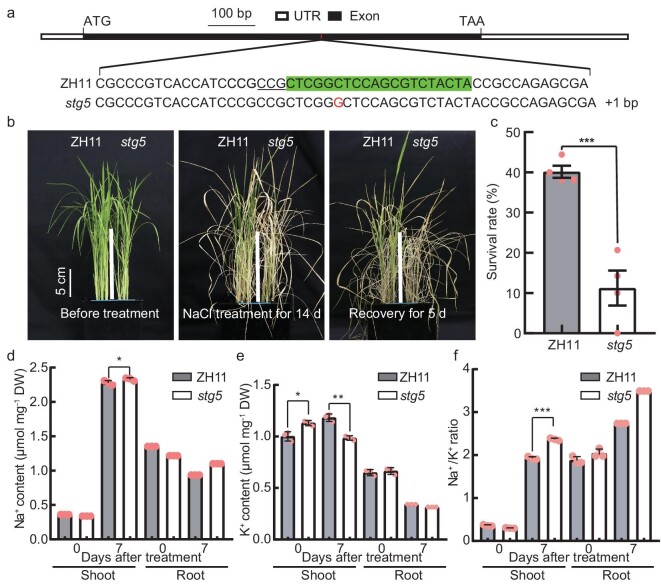
*STG5* is required for conferring salinity tolerance in rice. (a) Schematic diagram indicates the target and mutated site of *STG5* using CRISPR/Cas9 technology. The target sequence within the exon of *STG5* is highlighted using a box. Protospacer adjacent motif (PAM) sequence is shown as underlined. (b) The seedlings of ZH11 (Zhong Hua 11, the wild-type control) and *stg5* grown for 16 days (left panel), then transferred to 150 mM of NaCl for 14 days (middle panel), and recovered for 5 days under non-NaCl nutrient solutions (right panel). Scale bar, 5 cm. (c) Survival rate of ZH11 and *stg5* plants in (b) after recovery of 5 days. Data presented as mean ± SD; 24 plants were treated in each biological, and four biological replicates were performed. (d–f) The Na^+^ (d), K^+^ (e) contents and Na^+^/K^+^ ratio (f) in shoots and roots of ZH11 and *stg5* plants under normal (0 d) and salt-stress conditions (7 d). Data represent means ± SD. *P-*values were generated using Student's *t-*test. *P < 0.05; ***P < 0.001.

To verify the role of *STG5* in salinity stress tolerance, we generated the CRISPR knock-out line *stg5* containing a frame-shift mutation and key AP2 (APETALA2) domain disruption caused by 1 bp insertion (Fig. 4a and Fig. S12). The homozygous lines were subsequently employed for examining the phenotype under salinity stress conditions. The SR of *stg5* plants was only 0%–20%, while the SR of wild plants ranged from 38% to 44% under 150 mM of NaCl-stress conditions over 2 weeks, indicating that the *stg5* plants were more sensitive to salinity stress than the wild-type plants (Fig. 4b and c). Consequently, lower levels of chlorophyll were observed in *stg5* mutants compared with ZH11 plants after 7 days of treatment (Fig. S13). Previous studies have demonstrated that the homeostasis of Na^+^ and K^+^ is crucial for salt tolerance, and the Na^+^ contents in both the shoots and the roots of *stg5* and wild type (WT) plants were investigated under normal and NaCl-stress conditions. There was no clear difference in Na^+^ between *stg5* and WT plants under non-NaCl-stress conditions, whereas, under 150 mM of NaCl treatment for 7 days, the Na^+^ content was elevated in the shoots and roots of *stg5* (Fig. 4d). Additionally, we examined K^+^ contents within the same samples and found that there was higher or no obvious difference in shoots and roots of *stg5* plants under normal conditions. However, the K^+^ content in *stg5* mutants was significantly lower than that of wild-type plants following NaCl treatment (Fig. 4e). Consequently, the Na^+^/K^+^ ratio was either unchanged or lower under the non-NaCl conditions, while the Na^+^/K^+^ ratio was significantly increased in *stg5* plant shoots and roots following NaCl-stress treatment (Fig. 4f). This indicates that an imbalance of Na^+^ and K^+^ may contribute to hypersensitivity to salinity stress.

**Figure 5.**
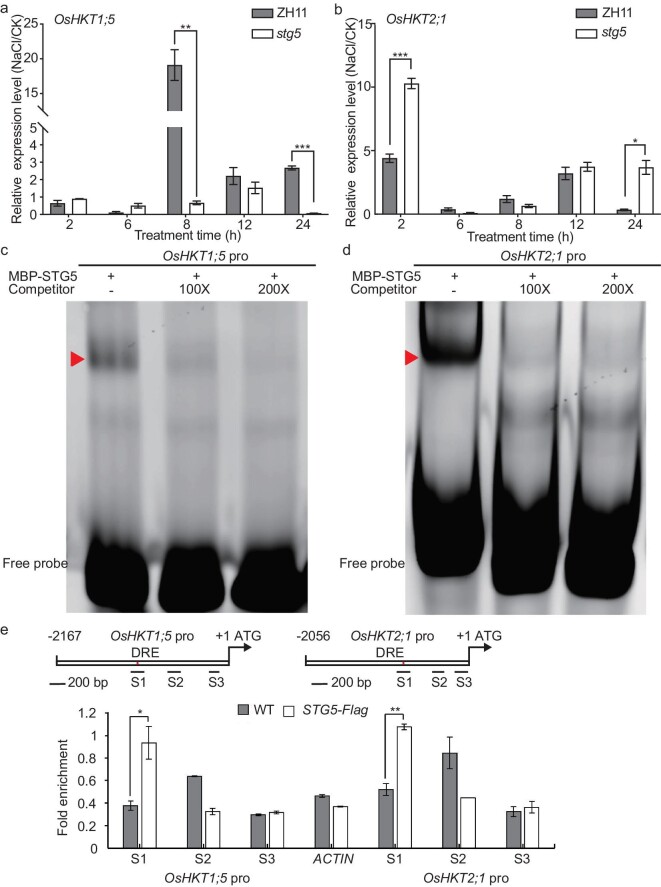
STG5 binds the promoters of *OsHKT1;5* and *OsHKT2;1* to maintain the Na^+^/K^+^ homeostasis. (a and b) The transcription levels of (a) *OsHKT1;5* and (b) *OsHKT2;1* in ZH11 and *stg5* seedlings with or without NaCl stress at multiple time points. Results from three technical replicates represented as means ± SE. Two biological replicates were performed and similar results were obtained. (c and d) DNA binding activity of STG5 on DRE motifs of (c) *OsHKT1;5* and (d) *OsHKT2;1* promoter fragments were tested using EMSA assay. Cold probes were used as competitors with indicated folds. The triangles indicate the shifted bands. (e) ChIP–qPCR assay indicating that the DNA fragments in the S1 region of *OsHKT1;5* and *OsHKT2;1* promoters that contain the DRE elements can be bound by STG5. The amplicon of *ACTIN* was taken as a negative control. The top scheme is a representation that illustrates the locations of the amplicons used for qPCR. DRE stands for the binding motif of the *STG5* transcription factor. *P-*values were generated using Student's *t*-test. *P < 0.05; **P < 0.01.

Given that *STG5* is essential for regulating the homeostasis of Na^+^ and K^+^, we were prompted to examine whether STG5 regulated ion transporters, such as the members of the *HKT* gene family. To address this, we assessed the transcription levels of *OsHKT1;1, OsHKT1;3, OsHKT1;4, OsHKT1;5, OsHKT2;1, OsHKT2;2, OsHKT2;3* and *OsHKT2;4* in *stg5* using qPCR and identified that their expression levels were significantly altered compared with wild-type plants (Fig. 5a and b, and Fig. S14a–f). This suggests that *STG5* modulated transcription either directly or indirectly. Notably, we found that the Na^+^ exporters of *OsHKT1;4* and *OsHKT1;5* were significantly reduced in *stg5* mutant plants compared with wild-type plants, while *OsHKT2;1*, the influx of the Na^+^ transporter, was strikingly upregulated in *stg5* plants (Fig. 5a and b, and Fig. S14c). Moreover, the expression level of high-affinity K^+^ transporter *OsHKT2;4* decreased in mutant plants (Fig. S14f). Based on this, the imbalance of Na^+^ and K^+^ homeostasis in the *stg5* mutants may be the primary cause of salt sensitivity. Subsequently, we performed *cis-*element scanning using the PlantCARE database [29] and noted that the promoters of *OsHKT1;5, OsHKT2;1, OsHKT2;3* and *OsHKT2;4* contain at least one DRE (dehydration-responsive element) core element (GCCGAC) that is the binding motif of the *ERF* (*ethylene response factor domain-containing transcription factors*) transcription factor. We then used EMSA (electrophoresis mobility shift assay) to determine whether STG5 could directly bind to the enriched region using MBP-STG5, indicating specific binding to the DRE element of the *OsHKT1;5, OsHKT2;1, OsHKT2;3* and *OsHKT2;4* promoters (a specific shifted bind indicated using arrows) (Fig. 5c and d, and Fig. S15a and b). The binding of STG5 to the promoter region was further reinforced through a ChIP (Chromatin immunoprecipitation) assay. The amplicons containing DRE elements were substantially enriched, while other regions were not enriched compared with the negative controls of the *actin* or *ubiquitin* promoter (Fig. 5e and Fig. S15c). This suggests that STG5 could directly bind the promoters of *OsHKT1;5, OsHKT2;1, OsHKT2;3* and *OsHKT2;4*. Collectively, our findings support the notion that *STG5* regulates the homeostasis of Na^+^ and K^+^ by modulating multiple members of the *OsHKT* gene family at the transcriptional level, conferring salt tolerance in rice.

## DISCUSSION

Plants frequently encounter abiotic stresses, including high salinity or drought stress, which severely constrain plant growth and result in yield loss. Many genetic or non-genetic factors affect the phenotype and phenotypic plasticity in response to environmental variation, and the regulations of transcription and mRNA abundance represent critical intermediate steps. This study focused on characterizing the sources of natural variation in gene expression responses to salinity stress events. Based on transcriptomic data under normal and salt-stress conditions, we constructed DEG and eGene data sets representing those responding to salt stress at the population level. Combining these data sets with GWAS analysis, we identified an *ERF* transcriptional factor (*STG5*), a novel and key salt-tolerance regulator, as well as its controlled signaling pathway of Na^+^/K^+^ homeostasis for the conferral of salt tolerance at the seedling stage. Our study will provide more key insights into the complex regulatory network of rice and a more effective method for determining crucial salt-stress regulators in the future.

eQTL mapping is a powerful tool for detecting genomic variations responsible for the regulation of transcriptome in crops. Recent studies have demonstrated that eQTL analysis is a more advantageous strategy for identifying drought and heat-stress-responsive genes [20,30]. Identifying salt-tolerance-associated eQTL will provide important clues for understanding the molecular mechanisms underlying salt adaptations in rice. It also lays the foundation for the selection and improvement of salt-tolerant rice varieties. We generated transcriptomic data following NaCl treatment for 24 hours from a Mini-Core panel of 202 rice accessions derived from different global regions, which have been used to construct a graph-based super pan-genome [24]. Compared with the transcriptome data under normal conditions, we obtained 12 898 DEGs and noted that 90 of 157 previously well-known and salt-related genes [27] exhibit significant enrichment in DEGs compared with all genes across the entire genome (*P* = 1.141e^−21^). This suggested that the DEGs data set provides a significant foundation for the future identification of salt-stress-associated genes.

Previous studies utilized 177 853 SNPs based on the transcriptome to identify eQTL only under normal conditions [18]. In this study, based on extensive genetic diversity (5 491 340 SNPs) of super pan-genome variation, a total of 49 955 eQTL were detected in normal and salinity stress environments according to the method [31] (Fig. 2a and b). Approximately 25% of the eQTL were static and identified across normal and NaCl-stress conditions (Fig. S5b). This indicates the conservation and functional importance of gene regulation and is valuable for optimizing stress-tolerant crop varieties. Remarkably, 17 461 of 29 657 dynamic eQTL were detected only under NaCl-stress conditions, capturing a copious number of regulatory variants for salt-stress response in rice seedlings. With respect to regulatory variants, we identified similar trends in terms of the proportion of *cis-* and *trans*-eQTL and the relative magnitude of effects in *cis* (Fig. S5b and c). However, relatively few meaningful *trans*-eQTL hotspots (214 under salt conditions, 107 under normal conditions) were characterized in our data. Among them, only a few well-known salt-related transcription factors (TFs) were identified in these hotspots. It is likely that our experiment had insufficient power to detect *trans-*eQTL. Considering the temporal, spatial and tissue-specific characteristics of the transcriptome, there are limitations associated with transcriptome analysis based on a 24-hour salt treatment, but it still reflects the early salt-stress response in rice, giving crucial clues for understanding and studying adaptation mechanisms. Utilizing time-series analysis can provide a more comprehensive and detailed understanding of salt-stress response, which is worthy of further investigation.

With higher-density genotype data as well as associated transcriptome data under different conditions, we utilized eQTL to determine the regulation of eGenes responding to salinity stress across the entire genome. Further analysis suggested that ∼47.5% (3696) and ∼45.6% (4268) of eGenes had only one unique eQTL under normal and salt-stress conditions, respectively. This is similar to most eGenes with one unique eQTL in other crops, such as cotton (∼67%) and maize (∼69%) [17,32] (Fig. 2c). Approximately 40.7% (in normal) and 43.4% (in salt-stress) eGenes were co-regulated by both *cis*- and *trans-*eQTL (Fig. 2d), suggesting the presence of complex regulatory networks of gene transcription under variable conditions. Moreover, we identified 3244 genes exhibiting differential responses to salt stress compared with normal conditions, which displayed enrichment in salt-inducible gene clusters (Fig. 2e). Additionally, 14 out of 157 well-known and salt-related genes pertain to both reGenes and salt-inducible gene clusters, demonstrating that there is a specific genetic correlation between reGenes and salt-inducible genes. These data sets may provide a gene pool for the future mining of salt-tolerant genes. Interestingly, the candidate gene *STG5* of the *qSTS5* peak belongs to reGene and salt-inducible gene clusters. Additionally, the neGenes and static eGene data sets are equally significant, such as the known salt-tolerant genes *OsHAK2* and *SKC1* belonging to neGenes and static eGenes, respectively (Fig. 2f). They provide comprehensive insights into understanding the mechanisms of rice salt-stress response and help reveal the complexity and diversity of gene regulatory networks.

Employing transcriptome data and eQTL results has been proven effective in assisting in the identification of candidate genes for phenotypic GWAS or QTL region identification [33]. Herein, we employed the GWAS for salt-tolerance-related traits at the seedling stage combined with transcriptomic data (DEGs and eQTL) to rapidly screen candidate genes linked to *STG5* (Fig. 3b and Table S3). Through gene function verification analysis, we identified that *STG5* is a novel salt-tolerance regulator to confer salt tolerance by maintaining the homeostasis of Na^+^/K^+^ (Figs 4 and 5). This indicates that transcriptome data at the population level after NaCl treatment are highly valuable for uncovering salt-related genes. Moreover, we found that the Hap1 variant of *STG5* exhibits stronger salt tolerance, which could be attributed to two factors: the critical variations in the promoter region leading to a differential salt-stress response between Hap1 and Hap2 or amino acid substitutions in the exon of *STG5* may affect its phosphorylation activity. After all, phosphorylation of serine is the archetypal post-translational modifications of proteins. Further research is required to comprehensively uncover the molecular mechanisms underscoring the differences in salt tolerance of the *STG5* Hap variant in the future, which will be beneficial for selecting and breeding salt-tolerant rice varieties. Additionally, the genetic relationship between *STG5* and *OsHKT1;5, OsHKT2;1, OsHKT2;3* or *OsHKT2;4* will be a fascinating question to fully explore in the future, which will be useful for fully exploring the underlying mechanism of *STG5* in regulating salt tolerance. Previous studies have named *LOC_Os05g49700* as *OsRPH1* (*REDUCED PLANT HEIGHT1*) and over-expression of *OsRPH1* resulted in a decrease in plant height and length of internode and leaf sheath, due to the reduced endogenous bio-active Gibberellins (GA) contents. These results suggested that *STG5* may be an important regulator for plant growth and salt-stress response.

## MATERIALS AND METHODS

Detailed materials and methods are available in the Supplementary information.

## Supplementary Material

nwae043_Supplemental_File

## Data Availability

RNA-seq raw data have been deposited in the NCBI SRA database with Bioproject number PRJNA1009219.
