# Supplementary material for: Uncovering key salt-tolerant regulators through a combined eQTL and GWAS analysis using the super pan-genome in rice
Source: Natl Sci Rev. 2024 Feb 5;11(4):nwae043. doi: 10.1093/nsr/nwae043 (PMC11034615; doi:10.1093/nsr/nwae043)
Supplement: nwae043_Supplemental_File [file nwae043_supplemental_file.docx]

**Supplementary information for**

### Uncovering key salt-tolerant regulators through a combined eQTL and GWAS analysis using the super pan-genome in rice

Hua Wei^1#^, Xianmeng Wang^1#^, Zhipeng Zhang^1#^, Longbo Yang^1#^, Qianqian Zhang^1#^, Yilin Li^1^, Huiying He^1^, Dandan Chen^1^, Bin Zhang^1^, Chongke Zheng^2^, Yue Leng^1^, Xinglan Cao^1^, Yan Cui^1^, Chuanlin Shi^1^, Yifan Liu^1^, Yang Lv^1,3^, Jie Ma^1,3^, Wenchuang He^1^, Xiangpei Liu^1^, Qiang Xu^1^, Qiaoling Yuan^1^, Xiaoman Yu^1^, Tianyi Wang^1^, Hongge Qian^1^, Xiaoxia Li^1^, Bintao Zhang^1^, Hong Zhang^1^, Wu Chen^1^, Mingliang Guo^1^, Xiaofan Dai^1^, Yuexing Wang^3^, Xiaoming Zheng^4^, Longbiao Guo^3^, Xianzhi Xie^2^*, Qian Qian^1,3,5^*, Lianguang Shang^1,5^*

**Address**

^1^Shenzhen Branch, Guangdong Laboratory of Lingnan Modern Agriculture, Genome Analysis Laboratory of the Ministry of Agriculture and Rural Affairs, Agricultural Genomics Institute at Shenzhen, Chinese Academy of Agricultural Sciences, Shenzhen, 518120, China

^2^ Institute of Wetland Agriculture and Ecology, Shandong Academy of Agricultural Sciences, Jinan, 250100, China.

^3^State Key Laboratory of Rice Biology, China National Rice Research Institute, Hangzhou, 310006, China

^4^National Key Facility for Crop Gene Resources and Genetic Improvement, Institute of Crop Science, Chinese Academy of Agricultural Sciences, Beijing 100081, China

^5^Yazhouwan National Laboratory, No. 8 Huanjin Road, Yazhou District, Sanya City, Hainan Province, 572024, China

^#^ These authors contributed equally to this work.

*Correspondence: [shanglianguang@caas.cn,](mailto:shanglianguang@caas.cn,) qianqian188@hotmail.com, xzhxie2010@163.com.

**This PDF file includes:**

Supplemental materials and methods

Figures 1 to 15

Tables 1 to 4

REFERENCES

**SUPPLEMENTAL MATERIALS AND METHODS**

**Plant materials, growth and phenotypic measurements**

A total of 202 rice accessions were selected from the super pan-genomic population to investigate the phenotypes under normal and NaCl stress condition [24]. Plump seeds were immersed in water at 37℃ for two days. And then, the germinated seeds were put in the bottomless plates and then were transferred to a hydroponic system. These seedlings were cultured with 1/4, 1/2 and whole Yoshida’s nutrient solutions for 16 days and were grown in greenhouse with 12 h light/12 h dark, 30±2℃ conditions. 16-day-old seedlings were transferred to 0 and 150 mM NaCl nutrient solution for 14 days. During this period, the nutrient solution was changed every three days. Then, the survival rate (SR) and dead leaf rate (DLR) were determined based on the criteria that plant with green leaves were considered survivable; bleached leaves with more than 2/3 of the leaf area were regarded as dead. The shoots and roots of six plants were sampled under normal and NaCl stress conditions respectively. And shoot fresh weight (SFW), shoot dry weight (SDW), and root fresh weight (RFW), root dry weight (RDW), root length (RL) was measured after treatment with 14 days or normal conditions. Three biological replicates were conducted, and each replicate of 24 plants were counted for measurement. The mutant plants of *STG5* (*LOC_Os05g49700*) and *m470* (*LOC_Os05g49470*) were generated by CRISPR/Cas9 technology according to the previous studies [1]. The single-guide RNAs (sgRNAs) listed in Supplementary Table 4. The sterilized seeds of Columbia (Col-0) and *atcor314* mutants were laid on Murashige and Skoog medium and grown under 12 h light/12 h dark, 22℃ conditions. After 7 days, these seedlings were transferred to MS medium contain 150 mM NaCl and cultured for 7 days. The roots lengths were measured by Image J software.

**Transcriptome sequencing**

For RNA-sequencing, the seedlings were grown under normal condition for 16 days, the leaves and shoots of three plants were sampled after 150 mM NaCl treatment with 24 hours. Total RNA was extracted using the TRIzol reagent (Invitrogen). RNA-seq library for each accession was constructed using the Truseq RNA Library Preparation kit, version 2. RNA samples were sequenced on the Illumina platform NovaSeq 6000 with a target read length of 150 bp.

**Reads mapping and differential expression analysis**

Sequenced reads of 202 rice accessions under NaCl stress condition were trimmed using Trimmomatic (Version 0.39) with parameters “ILLUMINACLIP: TruSeq3 PE.fa: 2:30:10,LEADING:3,TRAILING:3,SLIDINGWINDOW:4:15,MINLEN:36” [2]. Clean reads were mapped to the reference genome (MSU7.0) using Hisat2 (Version 2.2.1) [3]. RNA-seq data of the same genotypes under normal condition was obtained from our previous study [24 in main text]. Reads count of annotated gene were then calculated by featureCounts (Version 2.0.2) [4]. Differentially expressed genes (DEGs) among normal and salt conditions for each material were identified by the edgeR package [5], with the following parameters: bcv = 0.4, *P* < 0.01 and logFC ≥ 2 or ≤ -2. DEGs detected in at least 10% of the germplasms were retained, and further classified into three groups according to the proportion of samples: (class A, 20< n ≤ 101, 10~50%; class B, 101 < n ≤ 182, 50%~90%; class C, 182 < n ≤ 202, 90%~100%), n stands for the proportion of samples. GO analysis were then conducted in these groups using TBtools (V 1.120) with default parameters [6].

**Association analysis and eQTL identification**

Read counts of each gene was normalized to FPKM using Cufflinks with default parameters [7]. Genes with average FPKM > 0.1 were retained for the downstream analysis (23,736 and 26,450 genes expressed in normal and salt stress condition, respectively). The FPKM values were further normalized using quantile-quantile normalization (qqnorm) function in R (Version 3.1.2). After normalization, the top 20 hidden and confounding factors in the expression data, the normal quantile transformed expression value were calculated by using the probabilistic estimation of expression residuals (PEER) method [8]. The high-quality SNPs data was generated by our previous pan-genome research and complete assembly of the rice *Nipponbare* reference genome [24, 25 in main text]. A total of 5,491,339 SNPs with allele frequency > 0.05 and missing < 0.2 were filtered by VCFtools [9]. PCA was conducted to infer population structure with plinks (plink2 --pca 10). Both of the first 20 factors form PEER result and the first five principal components in PCA analysis were employed as covariates. eQTL analysis were performed by MatrixEQTL package (Version 2.2) [10]. The cutoff of *P=*1.82e^-7^ (1/n, where n indicates the number of SNPs) were used to filter associated other SNPs. The significant SNPs for each gene were grouped into one locus if the distance between adjacent SNPs were less than 200 kb. Only the loci with more than 20 significant SNPs were considered as candidate eQTLs, represented by the most significant SNPs (lead SNPs). Candidate eQTLs within a LD block (*r^2^* > 0.1, obtained from LDBlockShow with default parameters) were further merged into one locus, and the most significant eQTL was retained [11]. For each gene, eQTLs with lead SNP located within 200 kb upstream or downstream of the gene were classified as *cis*, while the others were regarded as *trans*. The *trans*-eQTL hotspots were identified by hot_scan software [12], with a window size of 20 kb and adjusted *P*-value < 0.01.

**Genome-wide association study**

The salt tolerance-related traits were measured. We performed GWAS used the linear mixed model in GEMMA (version gemma-0.98.5 -lmm -k -c). Plink (version v1.90b6.26) was used to perform PCA analysis (--pca 10) [13]. Kinship was conducted by GEMMA (-gk 2). The first five principal components matrix and kinship matrix as covariates. The horizontal dashed line shows the significant threshold of GWAS, which was calculated using Bonferroni correction and retained 4,627,345 SNPs for analysis (0.05/SNPs). Manhattan plot and QQ plot were created using CMplot package [14]. The SNPs with the lowest *P*-value in the one peak were identified to the lead SNPs. The colocalization of GWAS and eQTL results were analyzed by *coloc* (Version 5.2.2) r-package.

**Real-time PCR**

To investigate expression of the salt tolerance-associated genes, total RNA was extracted from leaves and shoots of plants in different time points (2, 6, 8, 12 and 24 hours) using the TRIzol reagent (Invitrogen). And reverse-transcribed into the fist-strand cDNA with a Kit (YEASEN, 11141ES60). Real time PCR was performed in the i-Cycler Bio-Rad machine, with each reaction containing 7.5 µL SYBR green MIX (YEASEN, 11201ES08), 3 µL cDNA that was diluted 10-folds, and 0.3 µL 10 mmol^-1^ primers in final volume of 15 µL. The PCR amplification program was 95℃ for 2 min, followed by 40 cycles of 95℃ 15 s, 55℃ for 15s, 72℃ for 15s. The *ACTIN* was used as the internal reference. The primers for qPCR were listed in Supplementary Table 4.

**Measurement of chlorophyll content**

The leaves of total chlorophyll content were measured as described previously [27 in main text]. The leaves of two weeks old seedling that treated with 150 mM NaCl for 7 days or non-NaCl stress condition were sampled and the fresh wights were recorded (W). Then, the leaves were soaked in 3 mL of 80% acetone (V/V) at room temperature for 48 hours. Absorbance was determined at 645 and 663 nm.

**Determination of Na^+^ and K^+^ content**

The concentration of Na^+^ and K^+^ were measured according to the method [15]. The shoots and roots were collected from ZH11 and *stg5* (three plants of each genotype) under normal condition and after NaCl treatment 7 days. Then the samples were washed clear and dried. The Na^+^ and K^+^ were extracted from the grounded samples and measured by flame photometer (AP1402).

**EMSA assay**

The coding sequence of *STG5* was fused with MBP and MBP-STG5 protein was extracted for subsequent EMSA assays. The probe was labeled with 5`6-FAM. Then, the labeled probes (0.5 μL of each) were incubated with purified protein in 20 μL reaction in room temperature for 1 h. After the reaction was complete, 2 μL of loading buffer was added to the reaction mixture and run the gel in 0.5 TBE buffer. The result was visualized by multispectral laser imaging system (Cytiva). The probes were listed in Supplementary Table 4.

**Chromatin immunoprecipitation (ChIP) PCR**

Transgenic plants and wildtype seedlings were sampled for ChIP analyses and performed based on the previous report [16]. The leaves were cross-linked with 1% (v/v) formaldehyde under vacuum for 30 min, and then ground with liquid nitrogen to extract the nuclear proteins, and sonicated to produce 250 bp DNA fragment for immunoprecipitation. The ChIPed DNA fragment were counted by quantitative PCR. The primers for ChIP-qPCR were listed in Supplementary Table 4.

**SUPPLEMENTAL FIGURES AND TABLES**


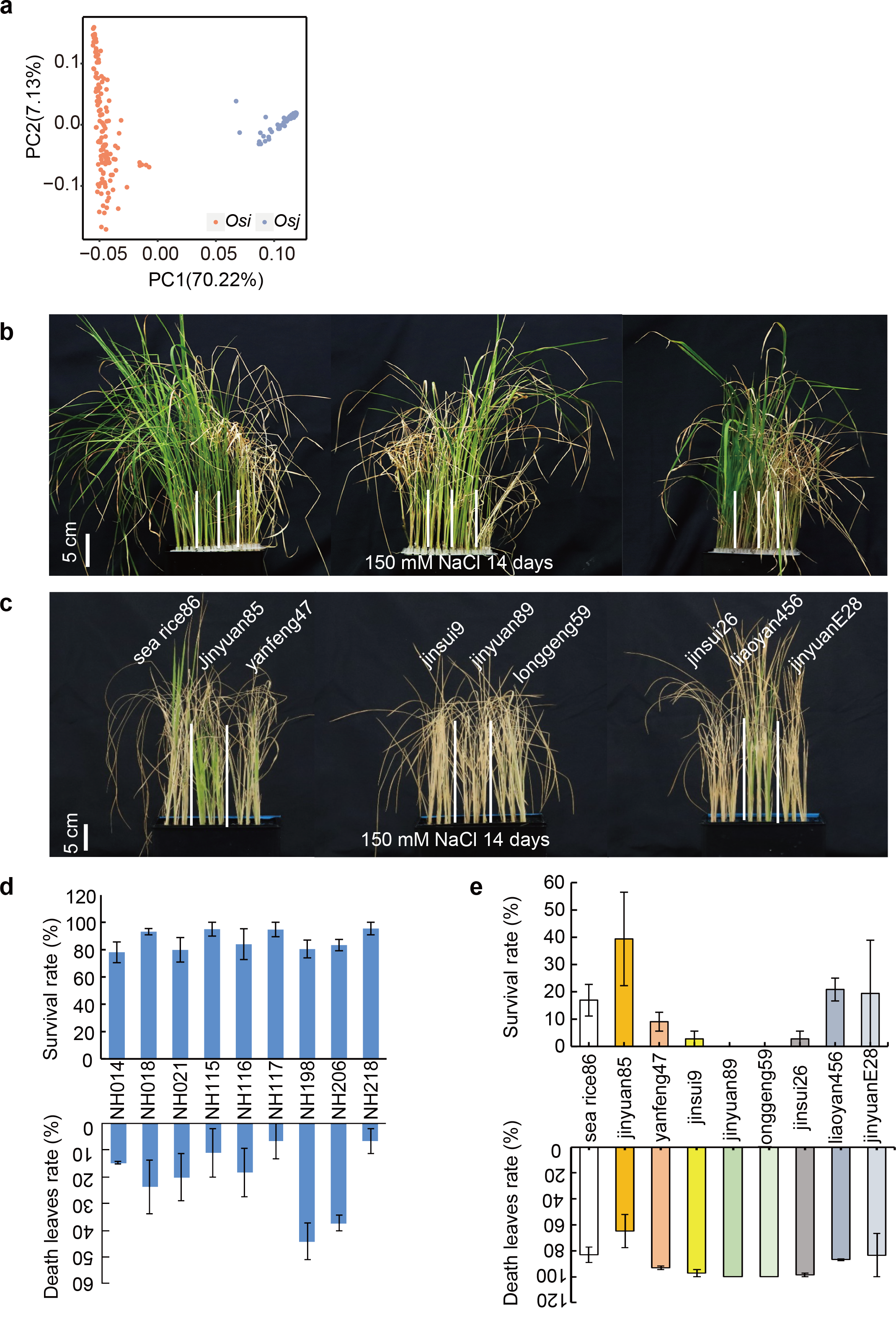


**Supplementary Fig. 1.** Salt tolerance diversity among different rice varieties in Mini-Core Collection.

1. Principal component analysis of all accessions: orange and blue points respectively represent *Indica* (*Osi*) and *Japonica* (*Osj*). (**b**) Salt response of 12 randomly selected rice varieties from 202 rice accessions. (**c**) The phenotypes of known salt tolerant cultivars under 150 mM NaCl treatment with 14 days. (**d-e**) Statistical analysis of the representative varieties of population (**d**) and the known salt tolerance cultivars (**e**) of survival rate (up) and dead leaf rate (down) after NaCl treatment with 14 days based on 24 plants. Data are presented as mean ± SD. n from three biological replicates.


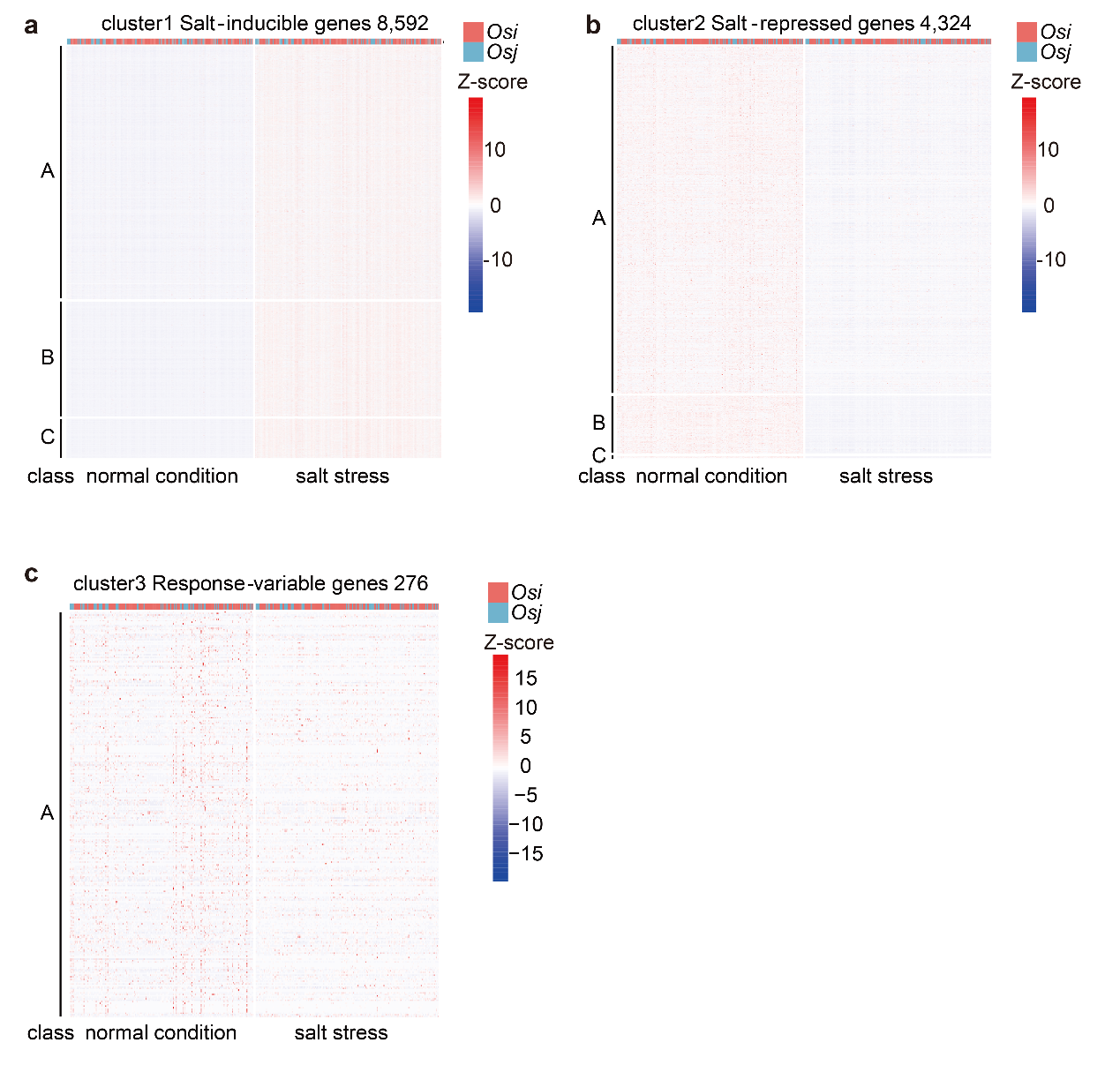


**Supplementary Fig. 2.** Expression pattern clustering 12,898 differentially expressed genes (DEGs) in response to salinity stress.

(**a-c**) The heat map exhibited variable salt-responsive patterns of three cluster genes. The FPKM values of each gene over all the samples were used. Cluster 1: genes were up-regulated in more than 20 varieties defined as salt-inducible genes (**a**); cluster2: genes were down-regulated in more than 20 varieties defined as salt-repressed genes (**b**); cluster3: responsive-variable genes existed both in cluster 1 and cluster 2 (**c**). Color of the bar (Z-score) indicated the FPKM values. The origin of rice accession was indicated by pink for *indica*, blue for *japonica*.


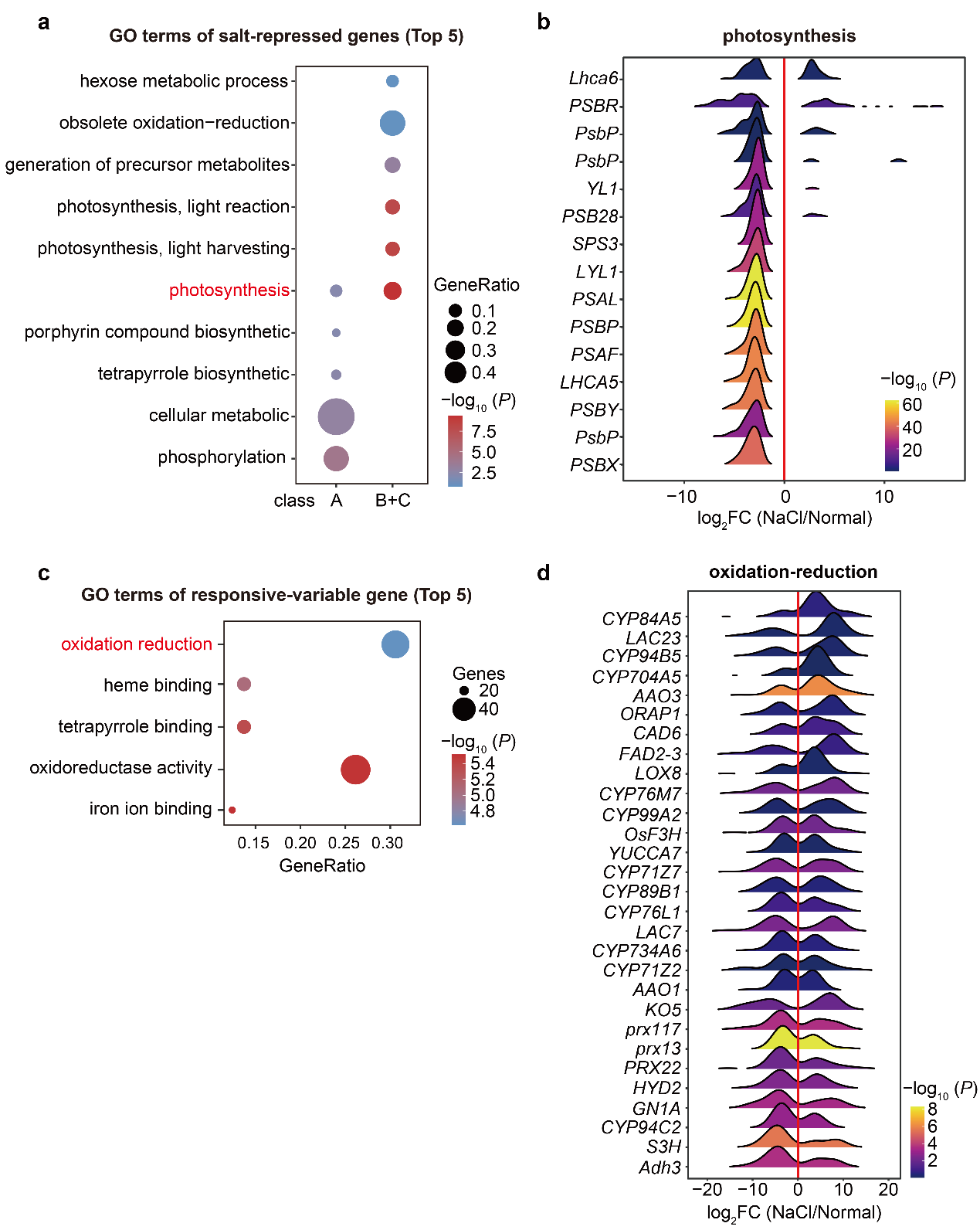


**Supplementary Fig. 3.** Salt-repressed and salt responsive-variable genes mainly involved in photosynthesis and oxidation reduction, respectively.

(**a, c**) Top five GO terms of salt-repressed genes (**a**), responsive-variable genes (**c**). (**b, d**) Distributed of log_2_ fold change of NaCl-to-control for photosynthesis of a highlighted by red letters (**b**), and the term of oxidative-reduction term of salt responsive-variable genes (**d**).


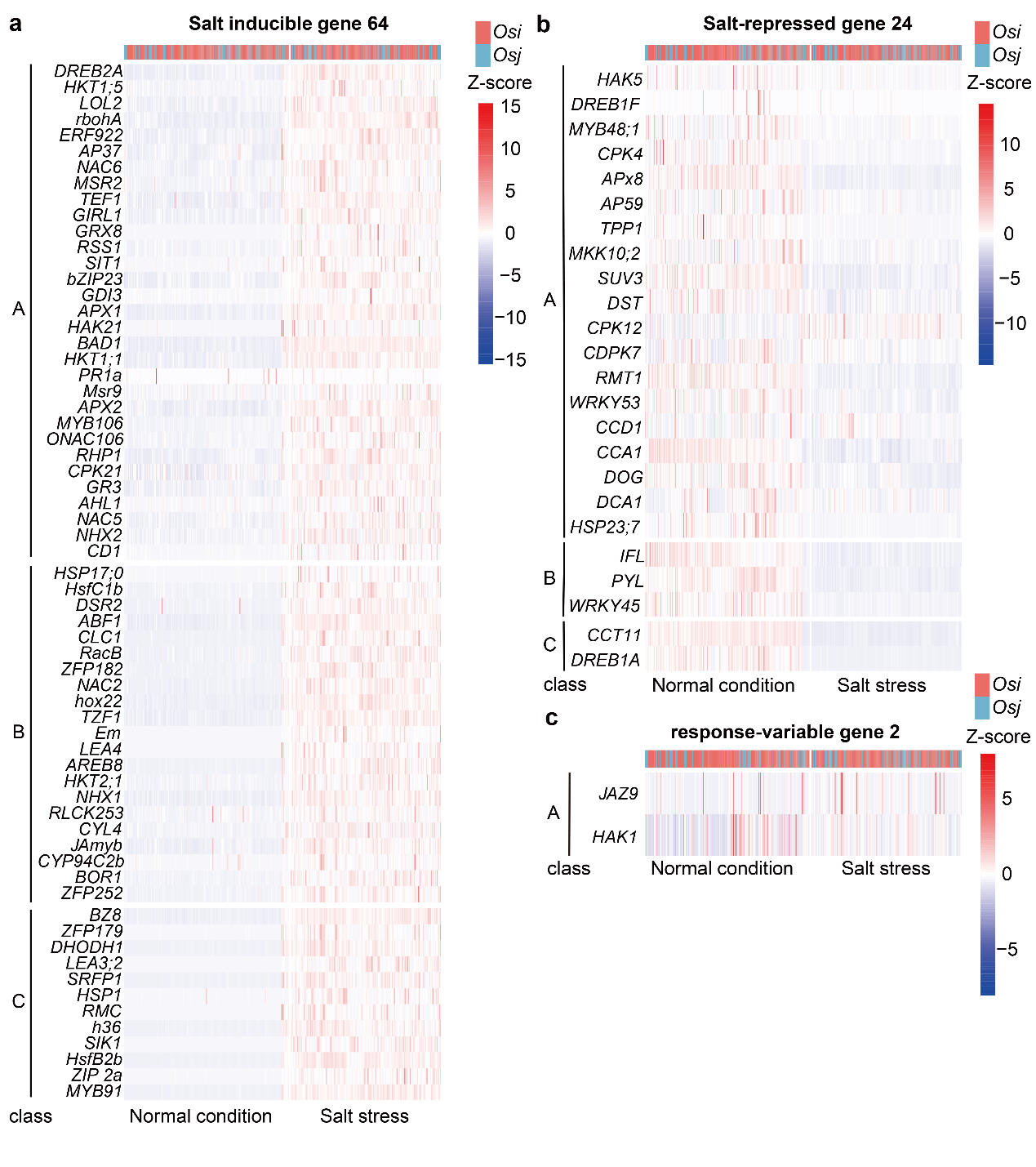


**Supplementary Fig. 4.** These known-salt responsive genes expression variations in response to the stress in the different accessions.

(**a-c**) Clustering analysis of the regulation pattern of known salt tolerance genes among 202 rice accessions. Among them, 62 genes classified into the salt-inducible genes cluster (**a**); 20 genes belonged to the salt-repressed genes cluster (**b**); *JAZ9* and *HAK1* classified into responsive variable genes cluster (**c**).


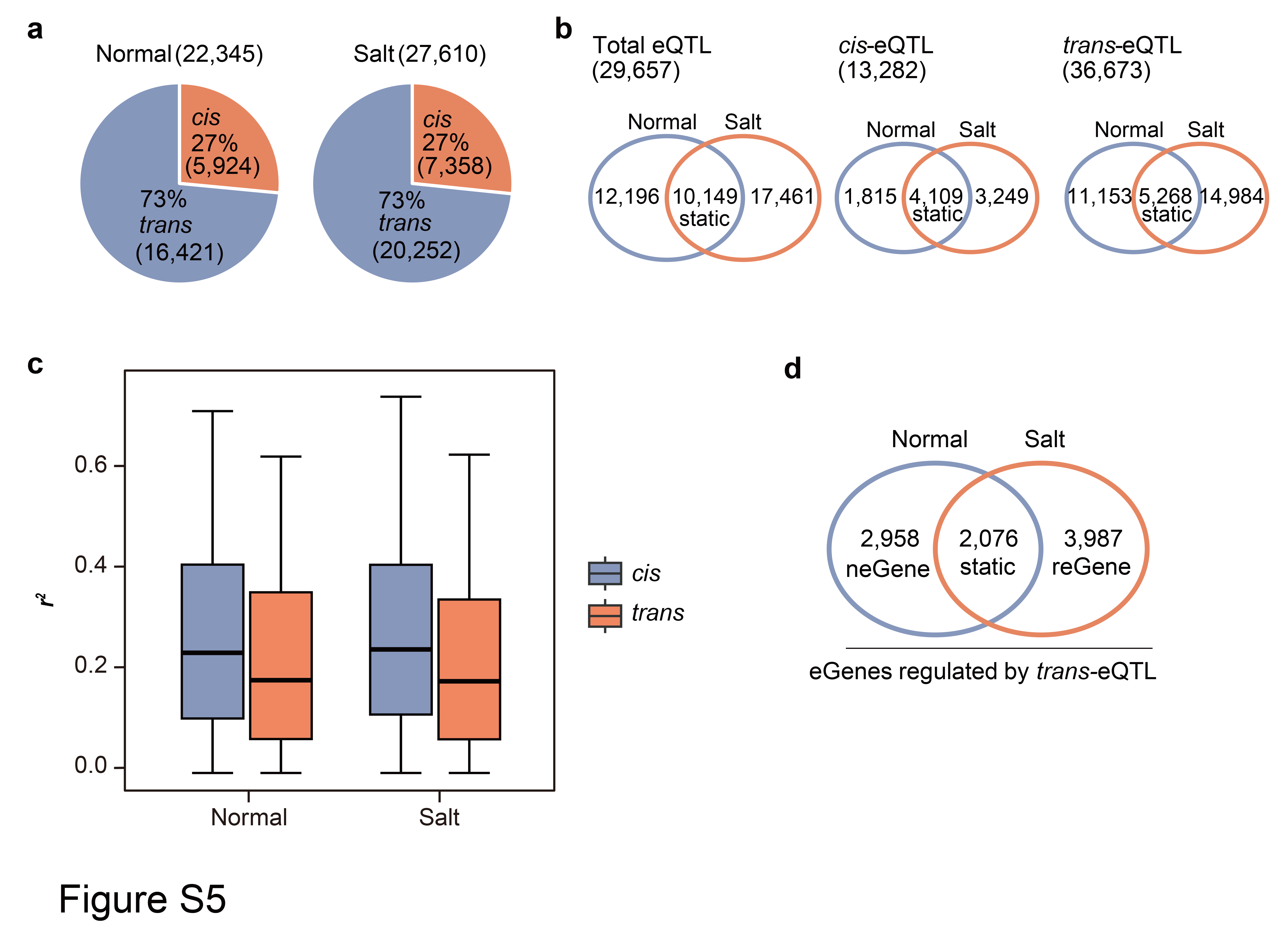


**Supplementary Fig. 5.** Large-scale *cis-* and *trans-*eQTLs identified by eGWAS.

(**a**) Percentage of *cis-* and *trans-*eQTL under normal and salt stress conditions, respectively. (**b**) The number of static and dynamic eQTLs in the total, *cis-* and *trans*-eQTLs. (**c**) The distribution of the effects of *cis-* and *trans*-eQTL. (**d**) Venn diagram showing the static and dynamic eGene (neGene and reGene represented normal and salinity stress conditions respectively) regulated by *trans-*eQTL.


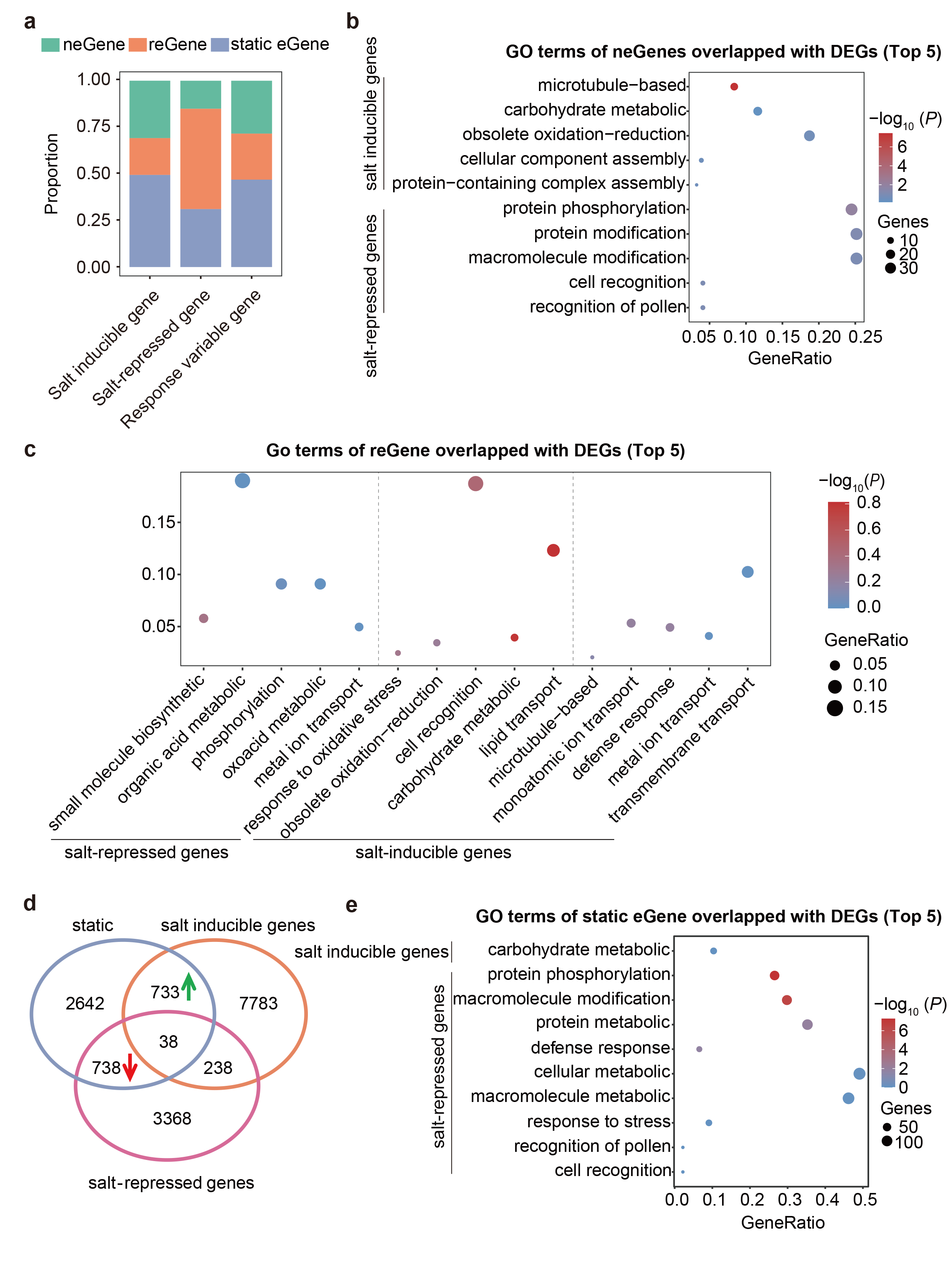


**Supplementary Fig. 6.** Association analysis of dynamic and static eQTLs with different expression genes in population level.

**(a)** Analysis the ratio of neGene, reGene and static eGene in salt inducible genes, salt-repressed genes and responsive genes respectively. (**b**) Top five GO terms of the sum of neGene overlapped with salt inducible and/or salt-repressed genes (1,091 genes) showed in figure 2h. (**c**) Top five GO terms of the sum of reGene overlapped with salt-inducible and/or salt-repressed genes (1,842 genes) showed in figure 2**g**. (**d**) Venn diagram showing static eGenes, salt-inducible and salt-repressed genes. (**e**) Top five GO terms of the sum of static eGene overlapped with salt-inducible and/or salt-repressed genes (1,747 genes) showed in **d**.


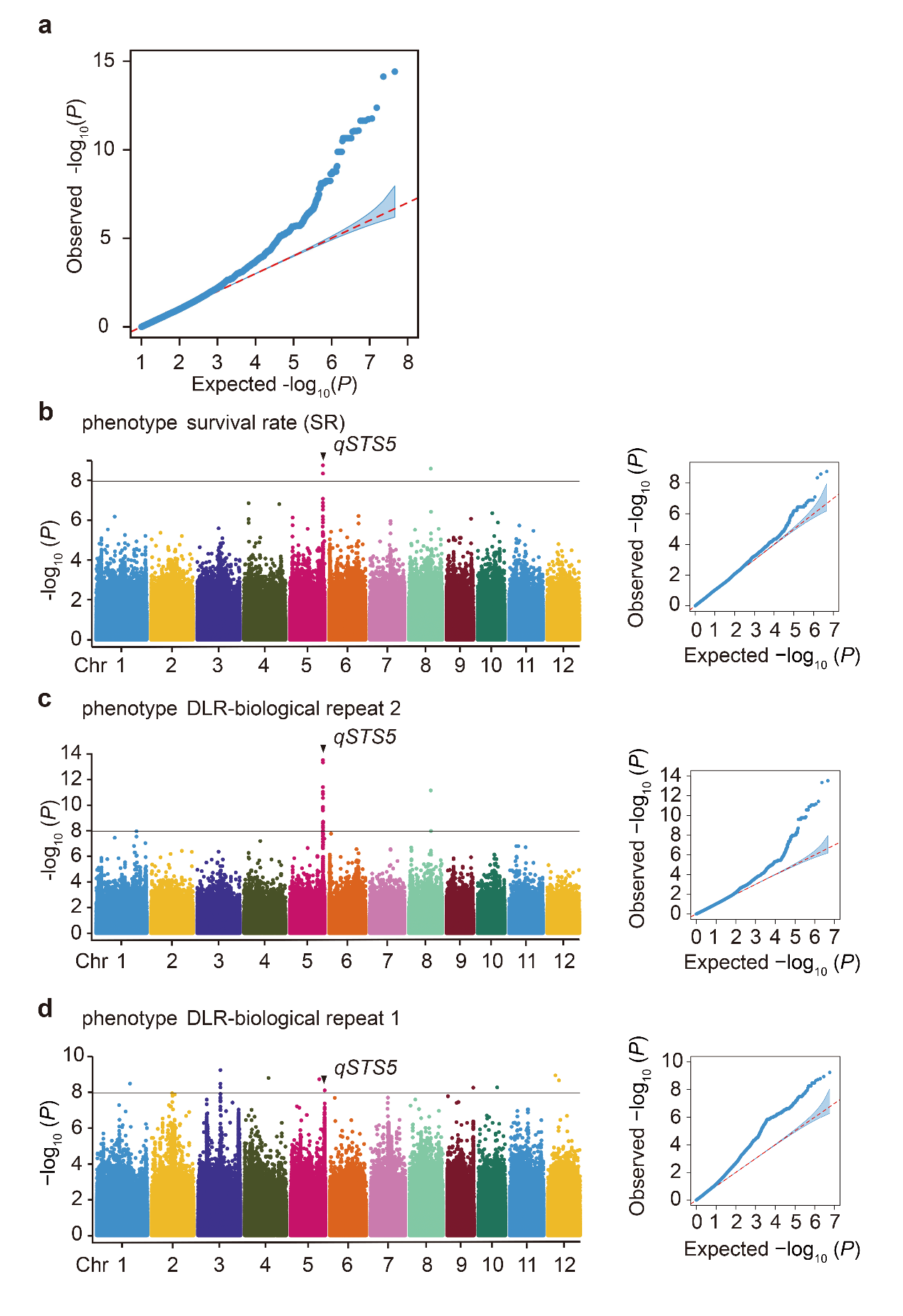


**Supplementary Fig. 7.** Manhattan plot of GWAS result showed the *qSTS5* site in different phenotypes.

1. Quantile-quantile (QQ) plots for GWAS based on the phenotype of salt tolerance level. (**b-d**) Manhattan and QQ plots for GWAS based on phenotype of survival rate (**b**), DLR biological repeat 1 (**c**) and 2 (**d**).


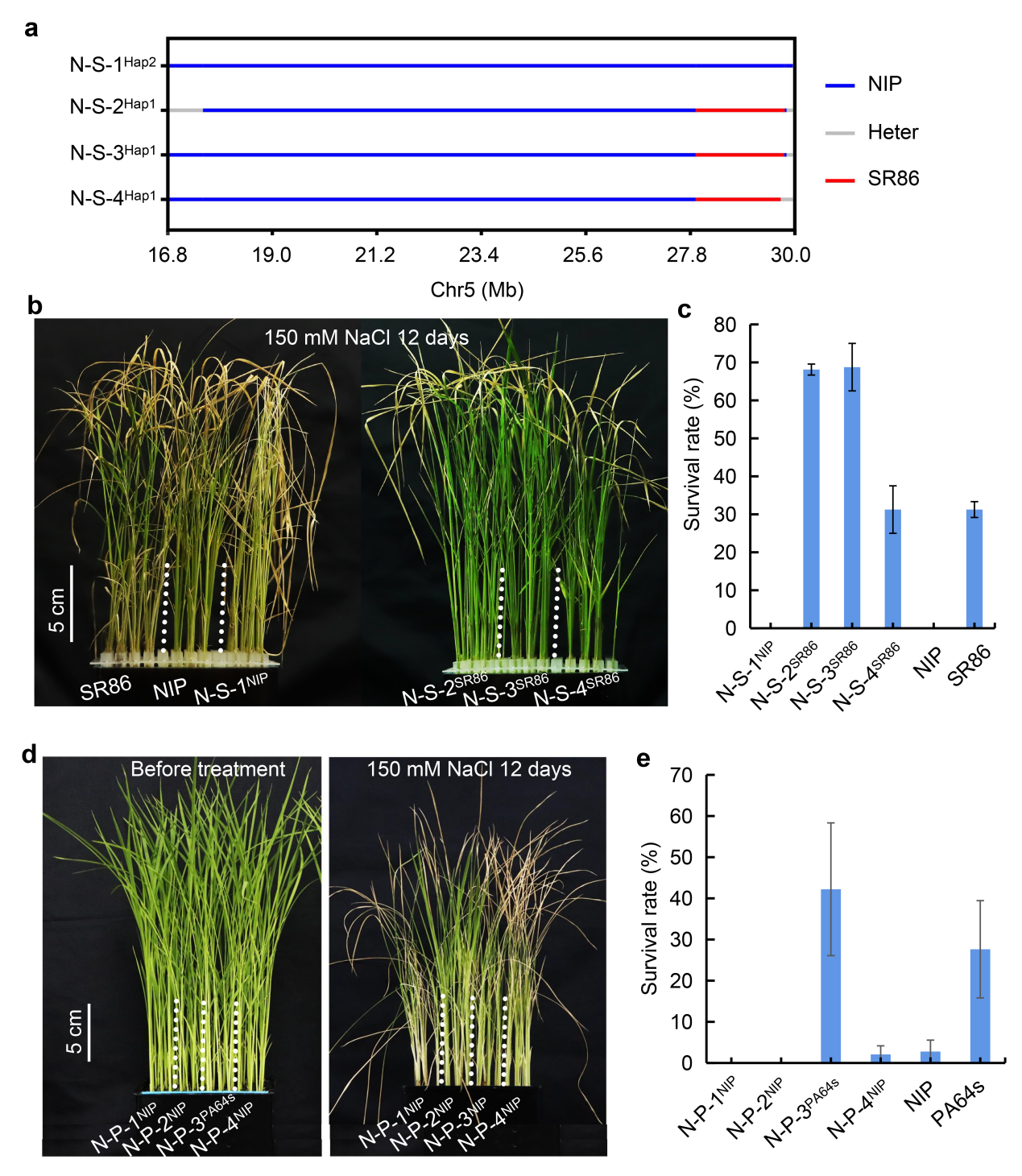


**Supplementary Fig. 8.** Phenotypes of CSSLs carrying the *qSTS5* site after NaCl treatment.

(**a**) The schematic drawing of CSSLs with substitution segments of SR86. The blue and red lines represented NIP and SR86 genomic region, respectively. (**b, d**) Phenotypes of CSSLs carrying and not carrying *qSTS5* sites at 12 days post 150 mM NaCl treatment. Scale bar, 5 cm. (**c, e**) Survival rates for plant materials in **b** and **d**, respectively.


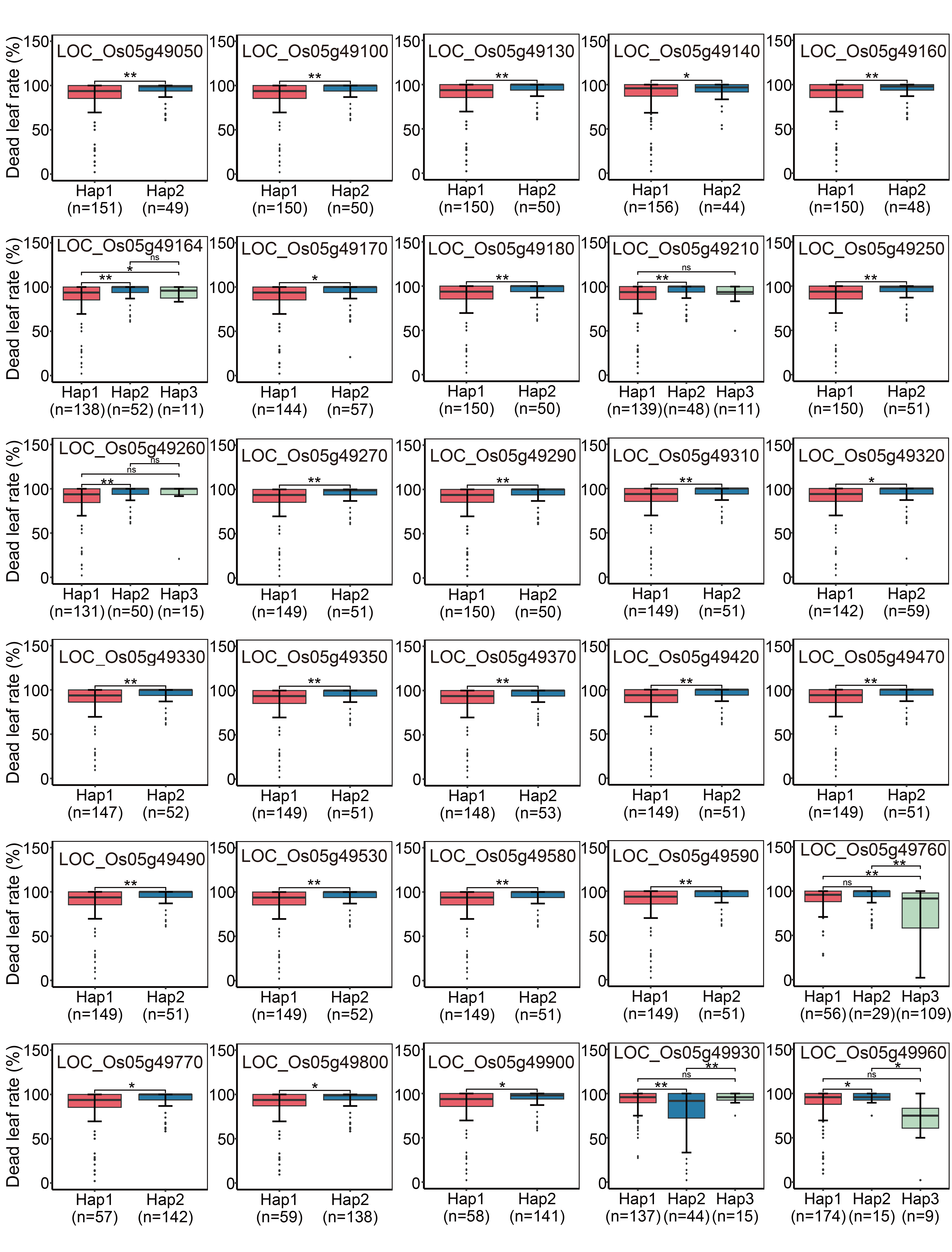


**Supplementary Fig. 9.** Divergence of dead leaf rate between different alleles of these candidate gene in 546.9 Kb genomic region. Error bars are SD, *P* value is determined by Student’s *t*-test, (*) *P<*0.05 and (**) *P<*0.01. ns stands for no significant with *P>*0.05.

**
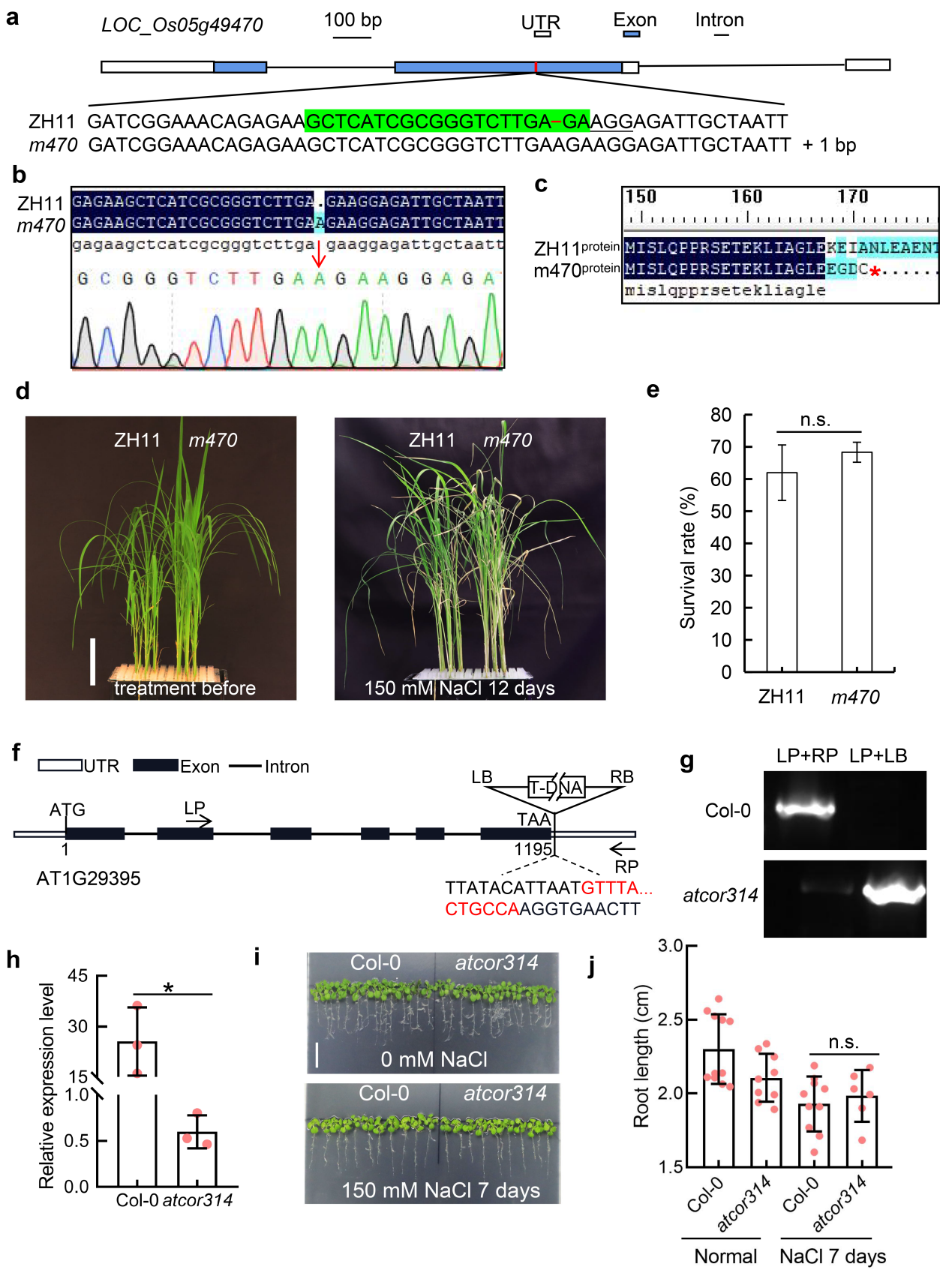
**

**Supplementary Fig. 10.** The phenotypes of *LOC_Os05g49470* and *AtCOR314* the homologous gene of *LOC_Os05g49170* under salt stress.

(**a**) Schematic diagram indicating the target and mutated site of *LOC_Os05g49470* by CRISPR/Cas9 technology. The target sequence within the second exon of *LOC_Os05g49470* and highlighted by green box. PAM sequence was shown as underline. (**b, c**) Sanger sequencing verified the mutation of *m470* that inserted one base resulting in a premature stop codon with 170 amino acid truncated protein. (**d**) The seedlings of ZH11 and *m470* grown for 14 days (left panel), then transferred to 150 mM NaCl for 12 days (right panel). Scale bar, 5 cm. (**e**) Survival rate of ZH11 and *m470* plants. Data presented as mean ± SE. (About 20 plants were treated in each of biological, and two biological replicates were performed). (**f**) The solitary homologous genes of *LOC_Os05g49170* in Arabidopsis (*AT1G29395, AtCOR314*) and the schematic diagram showing T-DNA insertion site in *atcor314* mutants. (**g, h**) The homozygous of *atcor314* mutants was identified by genomic PCR and RT-qPCR. (**i, j**) The phenotypes of Col-0 and *atcor314* with 150 mM NaCl treatment after7 days. (**j**) Statistical analysis of the root lengths of wild type plants and *atcor314* under normal and salt stress conditions. Data were presented as mean ± SD. *P* was generated by Student’s *t-*test with (*) *P*<0.05 and (n.s.) *P*>0.05.


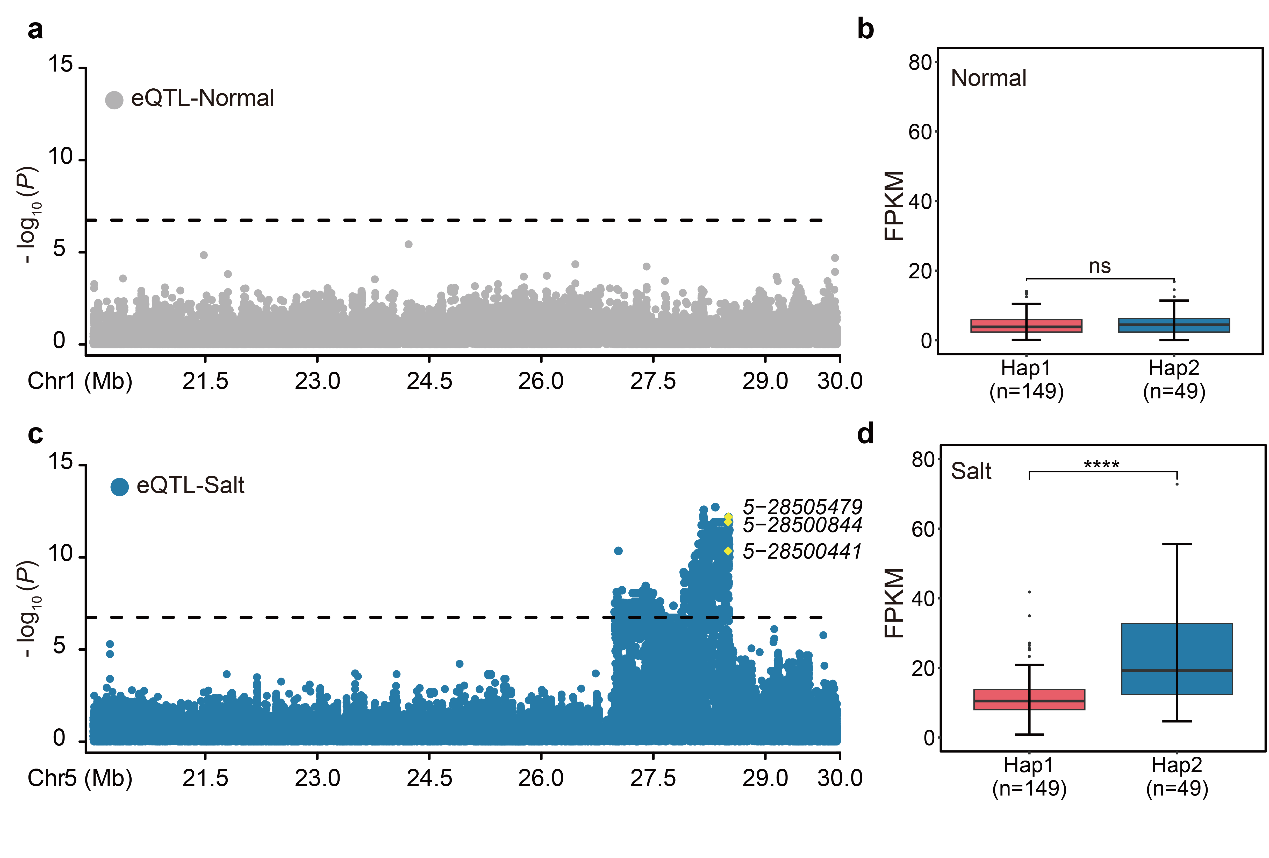


**Supplementary Fig. 11.** Association analysis of significant SNPs and haplotypes based on eQTL for candidate gene expression.

(**a, c**) The local Manhattan plots of eQTL for the most likely candidate gene of *LOC_Os05g49700* (named as *STG5*) under normal (**a**) and salt stress condition (**c**), respectively. The lead SNP (5-28,505,479), the four mutant sites in promoter (only 5-28,500,844 showed in figure, 28,500,857, 28,500,862, 28,500,881) and coding sequence (5-28,500,441) are presented as yellow dots. (**b, d**) Analysis of expression level (FPKM) under normal (**b**) and salt stress conditions (**d**) accessions with the identical genotypes of *STG5*. Error bar are SD, *P* value is determined by Student’s *t-*test. (****) *P*<0.00001, ns stands for no significant.


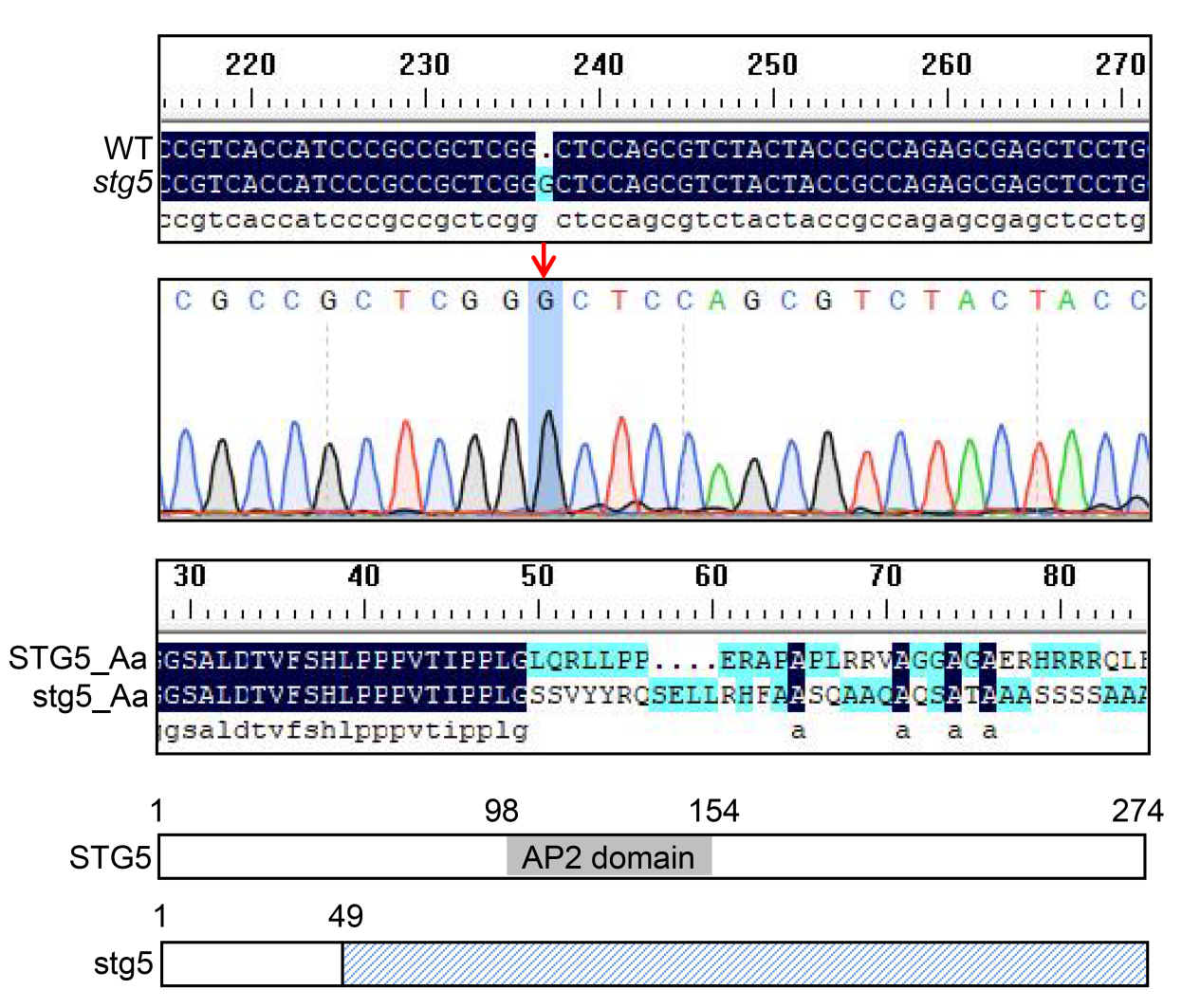


**Supplementary Fig. 12.** Characterization of *stg5* generated by genome editing. Sanger sequencing verified the mutation of *stg5* that insertion one base in the exon resulting in frameshift mutations.


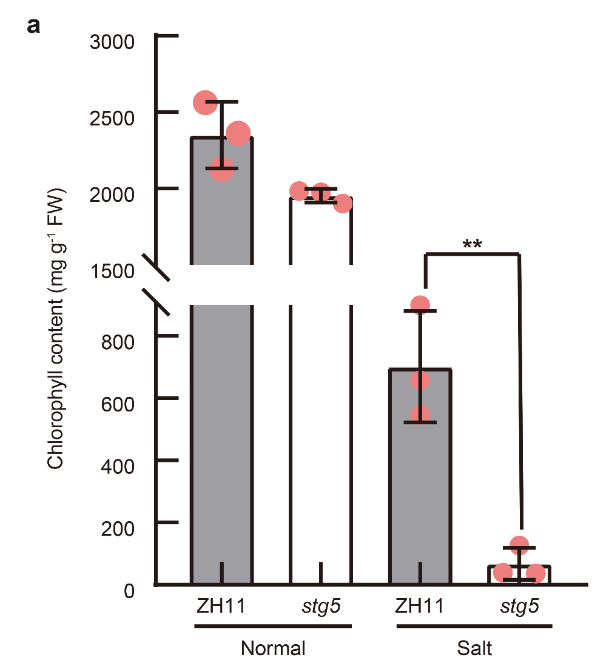


**Supplementary Fig. 13.** Statistical analysis of chlorophyll content of WT and *stg5* under non-NaCl and 150 mM NaCl for 7 days. Data presented as mean ± SD. Three biological replicates were conducted. *P* were generated by Student’s *t-*test, (**) *P*<0.01.


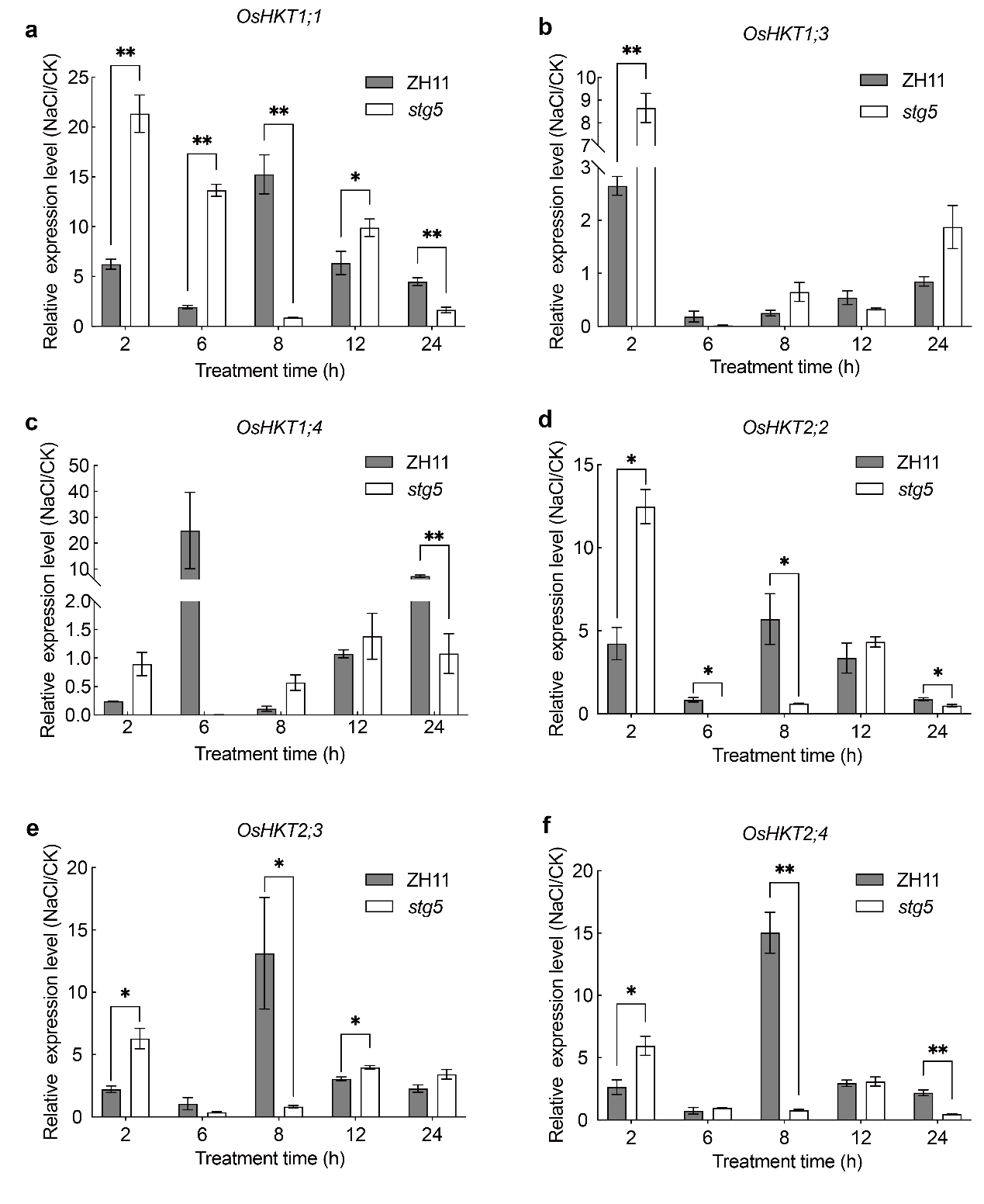


**Supplementary Fig. 14.** Transcription levels of *OsHKTs* members under normal and 150 mM NaCl conditions in different time treatment. Data from three technical replicates and represent as means ± SE. Two biological replicates were performed and obtained similar results. *P* value was generated by Student’s *t*-test, (*) *P<*0.05 and (**) *P<*0.01.


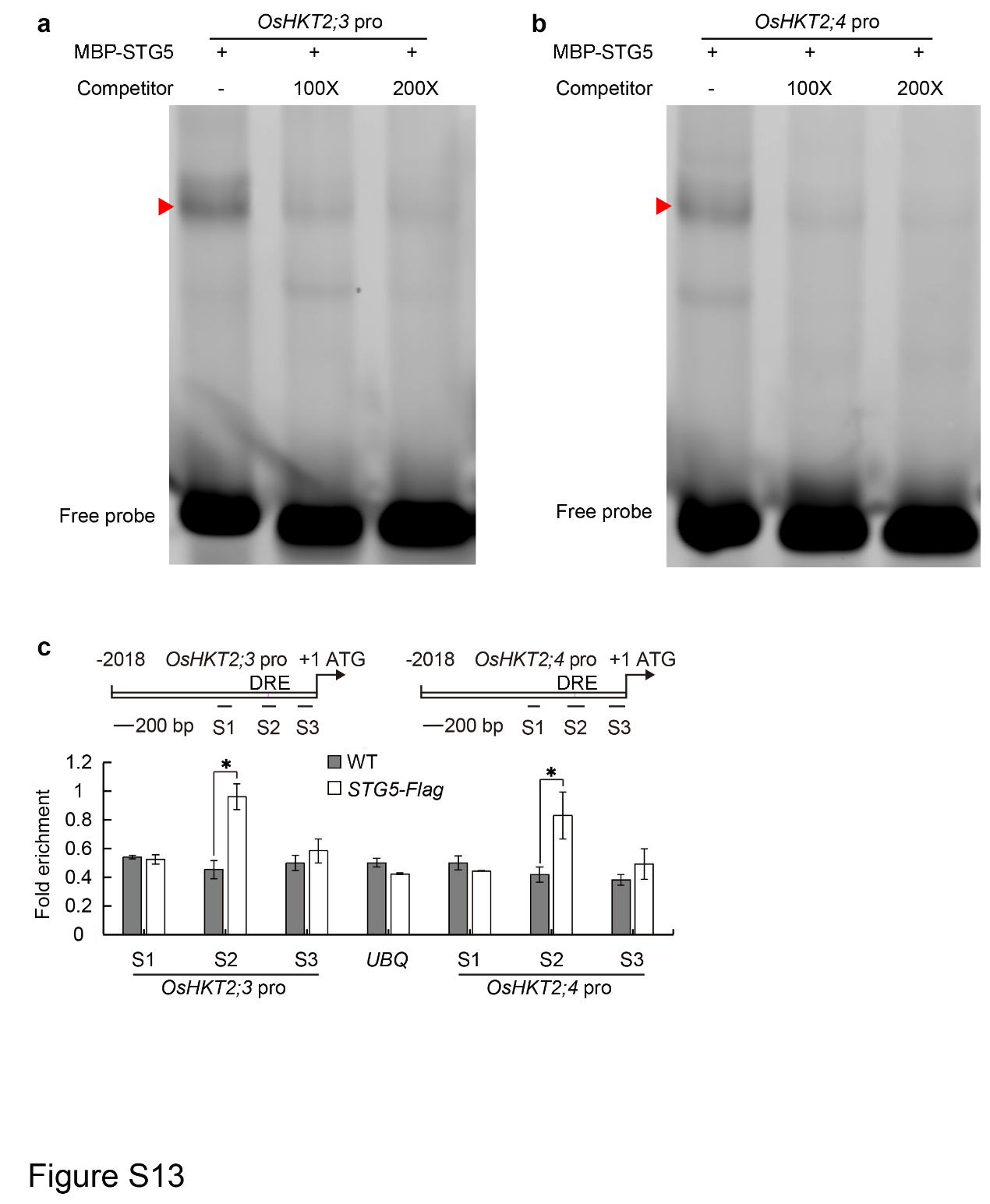


**Supplementary Fig. 15.** STG5 directly binds the promoters of *OsHKT2;3* and *OsHKT2;4*.

(**a, b**) EMSA assay showed that DNA binding activity of STG5 on *OsHKT2;3* (**a**) and *OsHKT2;4* (**b**) promoters. Cold probes were used as competitor with indicated folds. Red triangle indicates the shifted bands. (**c**) The enrichment on the promoter of on *OsHKT2;3* and *OsHKT2;4* tested by ChIP-qPCR assay. The top schemes were the representations that illustrated the locations of amplicons used for qPCR of *OsHKT2;3* and *OsHKT2;4* promoter, respectively. DRE stands for the binding motif of the *STG5* transcription factor. *P* value was generated by Student’s *t*-test, (*) *P<*0.05.

**Supplementary** **Table 1**. Phenotypic characteristics of eight salt-related traits under normal (N) or 150 mM NaCl (S) conditions among 202 rice accessions.


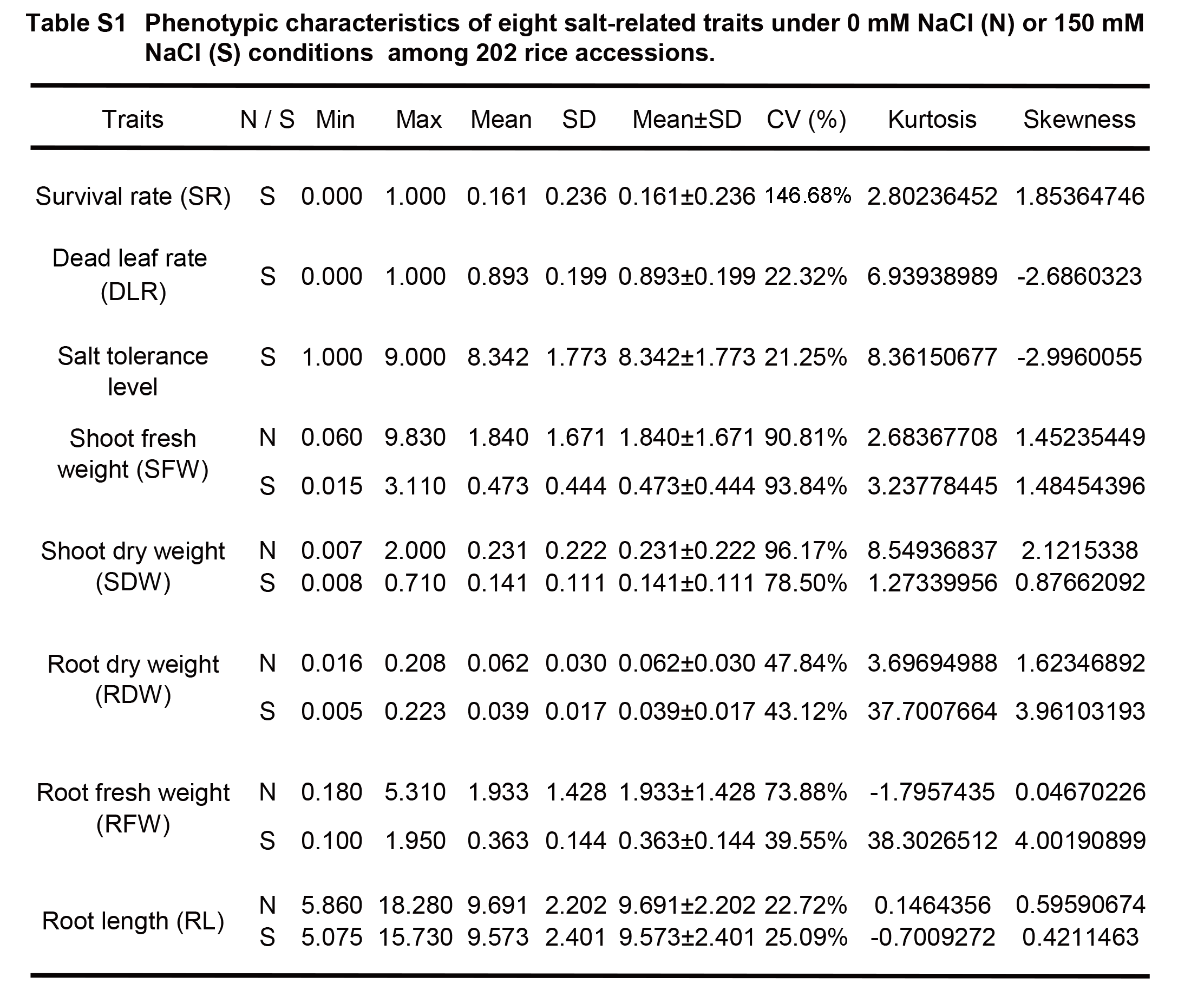


**Supplementary** **Table 2.** List of 28 salt associated loci that were uncovered by GWAS and based on eight salt tolerance traits.

| **Chr** | **Region (Mb)** | **Phenotype** | **Known site (yes or not)** | **Region of known site (Mb)** | **References** |
| --- | --- | --- | --- | --- | --- |
| 1 | 3.4-3.5 | STL | Yes | 2.8-4.4 | [17] |
| 1 | 42.1-42.2 | SFW_S | Yes | 39.7-43.0/  41.1-42.6 | [18]; [19] |
| 2 | 7.8-8.3 | STL | No |  |  |
| 2 | 10.8-10.9 | STL | Yes | 10.0-20.0 | [19] |
| 2 | 29.9-30.2 | STL | Yes | 23.4-30.0 | [20] |
| 3 | 15.5-15.8 | SR | No |  |  |
| 3 | 17.2-17.9 | SR | No |  |  |
| 3 | 18.1-18.2 | SR | No |  |  |
| 3 | 18.2-20.2 | DLR | No | 13.2-24.8 | [21] |
| 3 | 23.4-23.5 | SDW_S | No |  |  |
| 4 | 3.0-3.2 | SFW_S | No |  |  |
| 4 | 24.91-24.92 | DLR | Yes | 24.7-26.0 | [22] |
| 4 | 30.8-30.9 | DLR | Yes | 29.2-34.5 | [21] |
| 5 | 3.8-4.1 | SDW_S | No |  |  |
| 5 | 28.3-28.4 | SR | No |  |  |
| 7 | 6.8-7.2 | DLR | No |  |  |
| 7 | 19.92-19.95 | DLR | No |  |  |
| 8 | 18.8-18.9 | DLR | No |  |  |
| 9 | 1.2-1.7 | DLR | No |  |  |
| 9 | 5.8-9.3 | DLR | Yes | 8.6-9.0 | [23] |
| 9 | 10.8-10.9 | STL | No |  |  |
| 9 | 12.3-13.5 | DLR | No |  |  |
| 9 | 20.9-21.0 | STL | No |  |  |
| 9 | 21.4-21.5 | DLR | No |  |  |
| 11 | 7.62-7.63 | SFW_S | Yes | 5.7-9.0 | [17] |
| 11 | 22.22-22.24 | STL | No |  |  |
| 11 | 2.2-2.3 | DLR | Yes | 2.3-2.4 | [23] |
| 12 | 0.3-0.4 | STL | No |  |  |

**Supplementary Table 3**. Results of screening salt tolerance-related candidate genes in rice using *coloc* R package.


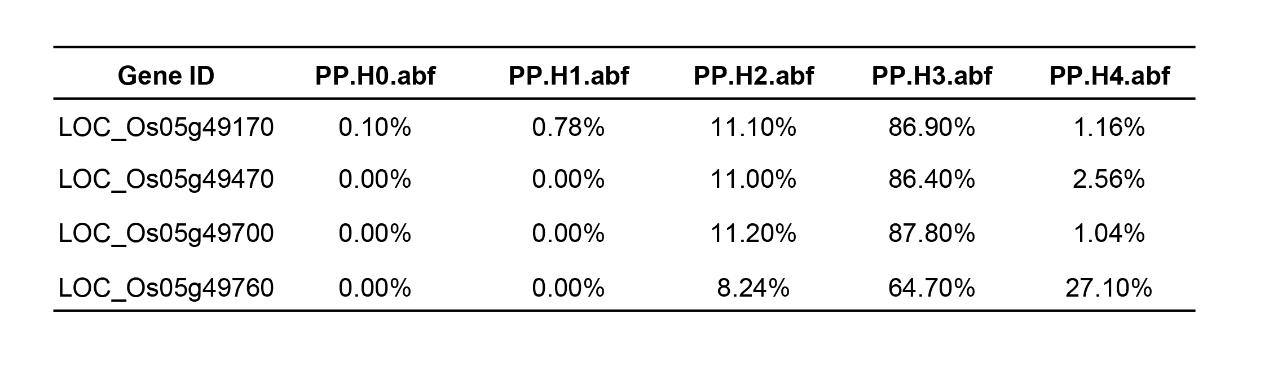


Note: PP.H0: the two traits of GWAS and eQTL in this region are not genetically correlated; PP.H1. and PP.H2: Only result of GWAS or eQTL exhibits a genetic correlation in this region, respectively; PP.H3.: Both traits are correlated, but the causal variables are different; PP.H4.: Both traits are correlated and share a common causal variable.

**Supplementary** **Table 4**. The primers list used in this study.

| **Prime name** | **sequence** |
| --- | --- |
| *STG5*-CRISPR-F | CTCGGCTCCAGCGTCTACTA |
| *STG5*-CRISPR-R | TAGTAGACGCTGGAGCCGAG |
| *m470-*CRISPR-F | GTGACTGTGAGAGGTTGTAC |
| *m470-*CRISPR-R | AACGTAGTTTTGTGTTGCGG |
| *LOC_Os05g49170*-qPCR-F | ACTATGCTGCTCGTGAGTGT |
| *LOC_Os05g49170*-qPCR-R | TGGTGAAGTGCTGAAAAGCG |
| *LOC_Os05g49470-*qPCR-F | TGCTAATTTGGAGGCCGAGA |
| *LOC_Os05g49470*-qPCR-R | GTGCTCATGTTCTGCTGCTCT |
| *LOC_Os05g49700*-qPCR-F | GAAATCTCGAGCAAATCACCAC |
| *LOC_Os05g49700*-qPCR-R | GCGGGAGGGTAATTTCGTAATT |
| *OsHKT1;1*-qPCR-F | TTCACCACTCTTGCGGCTATG |
| *OsHKT1;1*-qPCR-R | TGTTTGTAGCCAGTCTCCCCAG |
| *OsHKT1;4*-qPCR-F | GTCGAAGTTGTCAGTGCATATGG |
| *OsHKT1;4*-qPCR-R | TGAGCCTCCCAAAGAACATCAC |
| *OsHKT1;3-*qPCR-F | CCTCTCTTCAGCAATCCTAGTC |
| *OsHKT1;3*-qPCR-R | CGGTCTTAGAATCCTCACCATC |
| *OsHKT2;2*-qPCR-F | CTGCTGGTTCTCTGGTACATAAG |
| *OsHKT2;2*-qPCR-R | GTGACCGAGAATGAGAAGAGTG |
| *OsHKT2;3*-qPCR-F | GCCTCCTGTTGCTACTCATT |
| *OsHKT2;3*-qPCR-R | CTTAGTCCTCTCAATGCCCATATC |
| *OsHKT2;4*-qPCR-F | CTCCCTATGTTTCTGAGGTTGG |
| *OsHKT2;4*-qPCR-R | CCTACTGAGCTGCTGTTGTT |
| *ACTIN*-qPCR-F | TGGCATCTCTCAGCACATTCC |
| *ACTIN*-qPCR-R | TGCACAATGGATGGGCCAGA |
| *Ubq*-qPCR-F | CTTTCAGCTGAGGCCCAAGA |
| *Ubq*-qPCR-R | ACGATTGATTTAACCAGTCC |
| *OsHKT1;5*-S1-F | TAGCCGGTGGTGGCAGATACAC |
| *OsHKT1;5*-S1-R | CCCTAATCGATAGGCGATGACA |
| *OsHKT1;5*-S2-F | GGTTCCTGGTTCCTGCTACAAG |
| *OsHKT1;5*-S2-R | CATATATATCCTAAAAGCTTCA |
| *OsHKT1;5*-S3-F | ACGAGTGCCCTTGGTGCAATAG |
| *OsHKT1;5-*S3-R | TGTGGGGTTACCAGGTGCTGTG |
| *OsHKT2;1*-S1-F | TCGAAATGCTTGCCGATCAATG |
| *OsHKT2;1-*S1-R | CCAAGTTATGCTGACCTGATTG |
| *OsHKT2;1*-S2-F | ACGCTGAGCAGGTAATCATTAG |
| *OsHKT2;1*-S2-R | CTGATACTACCCATGGATGACT |
| *OsHKT2;1*-S3-F | AGTTATACCGGTCTTACTGTCC |
| *OsHKT2;1*-S3-R | GCAACGAGCCAACGAGTATGAG |
| *OsHKT2;3*-S1-F | AGTCCAGAAATGCTTGTCCACT |
| *OsHKT2;3*-S1-R | TGGACAGAAATCGTTCCTCTAG |
| *OsHKT2;3*-S2-F | CCATATCATCTCAGAAGTACTC |
| *OsHKT2;3*-S2-R | CAGGCAGGCATGCTCCTCAACC |
| *OsHKT2;3*-S3-F | ACCTCAACCATGTTGCACATGT |
| *OsHKT2;3*-S3-R | TCTTGATTCAAGATGGAACTGC |
| *OsHKT2;4*-S1-F | GACGAGTCTAGAGTCCATATAG |
| *OsHKT2;4*-S1-R | CGAAACTACTAATGTGTAGTATTC |
| *OsHKT2;4*-S2-F | ATCACCATATCATTCCAGATGT |
| *OsHKT2;4*-S2-R | GCAGGCAGGCATGCTCCTCAAC |
| *OsHKT2;4*-S3-F | CCTCCTCTCCACTAATATTAAC |
| *OsHKT2;4*-S3-R | GGCAGAGCCTCTAGCTCCTTAT |
| *OsHKT1;5pro*-FAM-F | CGATGACAAGAGCGGCCGACAGTACATTAGCTGGAGGGAGCCGACACTTGATCAGGTGGC |
| *OsHKT1;5pro*-FAM-R | GCCACCTGATCAAGTGTCGGCTCCCTCCAGCTAATGTACTGTCGGCCGCTCTTGTCATCG |
| *OsHKT1;5pro*-cold-F | CGATGACAAGAGCGGCCGACAGTACATTAGCTGGAGGGAGCCGACACTTGATCAGGTGGC |
| *OsHKT1;5pro*-cold-R | GCCACCTGATCAAGTGTCGGCTCCCTCCAGCTAATGTACTGTCGGCCGCTCTTGTCATCG |
| *OsHKT2;1pro*-FAM-F | TACCCGAGAACAAAGCACGGTATGCTGACCGAGAAGAATACTCCCTTGAGGACTCCCACC |
| *OsHKT2;1pro*-FAM-R | GGTGGGAGTCCTCAAGGGAGTATTCTTCTCGGTCAGCATACCGTGCTTTGTTCTCGGGTA |
| *OsHKT2;1pro*-cold-F | TACCCGAGAACAAAGCACGGTATGCTGACCGAGAAGAATACTCCCTTGAGGACTCCCACC |
| *OsHKT2;1pro*-cold-R | GGTGGGAGTCCTCAAGGGAGTATTCTTCTCGGTCAGCATACCGTGCTTTGTTCTCGGGTA |
| *OsHKT2;3pro*-FAM-F1 | CTCCTCATTCACAAATGGAGGGTTATTGCCGACAAATGAGAGTATGGTTGTATTCTCCTC |
| *OsHKT2;3pro*-FAM-R1 | GAGGAGAATACAACCATACTCTCATTTGTCGGCAATAACCCTCCATTTGTGAATGAGGAG |
| *OsHKT2;3pro*-cold-F1 | CTCCTCATTCACAAATGGAGGGTTATTGCCGACAAATGAGAGTATGGTTGTATTCTCCTC |
| *OsHKT2;3pro*-cold-R1 | GAGGAGAATACAACCATACTCTCATTTGTCGGCAATAACCCTCCATTTGTGAATGAGGAG |
| *OsHKT2;4pro*-FAM-F | GAAATTCTTGGCAATTGGTATAACATTGCCGACATCCGAAGGTTAATAGAGTTATTACAG |
| *OsHKT2;4pro-*FAM-R | CTGTAATAACTCTATTAACCTTCGGATGTCGGCAATGTTATACCAATTGCCAAGAATTTC |
| *OsHKT2;4pro*-cold-F | GAAATTCTTGGCAATTGGTATAACATTGCCGACATCCGAAGGTTAATAGAGTTATTACAG |
| *OsHKT2;4pro*-cold-R | CTGTAATAACTCTATTAACCTTCGGATGTCGGCAATGTTATACCAATTGCCAAGAATTTC |
| *atcor314*-LP | TGTTTCTAGGTTATCGGGTG |
| *atcor314*-RP | TCAGCTTTTAAATATCCCAATCC |
| T-DNA-LB | ATTTTGCCGATTTCGGAAC |
| *AtCOR314*-qPCR-F | GCTTAGGTTATCGGGTGAT |
| *AtCOR314*-qPCR-R | CGAATAGTGGAACAACAACTG |
| *AtACTIN2*-qPCR-F | GCTGAGAGATTCAGATGCCCA |
| *AtACTIN2*-qPCR-R | GTGGATTCCAGCAGCTTCCAT |

**REFERENCES**

1 Ma, X. *et al.* A Robust CRISPR/Cas9 System for Convenient, High-Efficiency Multiplex Genome Editing in Monocot and Dicot Plants. *Mol. Plant* **8**, 1274-1284 (2015).

2 Bolger, A. M., Lohse, M. & Usadel, B. Trimmomatic: a flexible trimmer for Illumina sequence data. *Bioinformatics* **30**, 2114-2120 (2014).

3 Kim, D., Paggi, J. M., Park, C., Bennett, C. & Salzberg, S. L. Graph-based genome alignment and genotyping with HISAT2 and HISAT-genotype. *Nat. Biotechnol.* **37**, 907-915 (2019).

4 Liao, Y., Smyth, G. K. & Shi, W. featureCounts: an efficient general purpose program for assigning sequence reads to genomic features. *Bioinformatics* **30**, 923-930 (2014).

5 Robinson, M. D., McCarthy, D. J. & Smyth, G. K. edgeR: a Bioconductor package for differential expression analysis of digital gene expression data. *Bioinformatics* **26**, 139-140 (2010).

6 Chen, C. *et al.* TBtools: an integrative toolkit developed for interactive analyses of big biological data. *Mol. Plant* **13**, 1194-1202 (2020).

7 Trapnell, C. *et al.* Transcript assembly and quantification by RNA-Seq reveals unannotated transcripts and isoform switching during cell differentiation. *Nat. Biotechnol.* **28**, 511-515 (2010).

8 Stegle, O., Parts, L., Piipari, M., Winn, J. & Durbin, R. Using probabilistic estimation of expression residuals (PEER) to obtain increased power and interpretability of gene expression analyses. *Nat. Protoc.* **7**, 500-507 (2012).

9 Danecek, P. *et al.* The variant call format and VCFtools. *Bioinformatics* **27**, 2156-2158 (2011).

10 Shabalin, A. A. Matrix eQTL: ultra fast eQTL analysis via large matrix operations. *Bioinformatics* **28**, 1353-1358 (2012).

11 Dong, S. S. *et al.* LDBlockShow: a fast and convenient tool for visualizing linkage disequilibrium and haplotype blocks based on variant call format files. *Brief. Bioinform.* **22**, bbaa227 (2021).

12 Silva, I. T., Rosales, R. A., Holanda, A. J., Nussenzweig, M. C., Jankovic, M. Identification of chromosomal translocation hotspots via scan statistics. *Bioinformatics* **30**, 2551-2558 (2014).

13 Chang, C. C. *et al.* Second-generation PLINK: rising to the challenge of larger and richer datasets. *GigaScience* **4**, 7 (2015).

14 Yin, L. *et al.* rMVP: A Memory-efficient, Visualization-enhanced, and Parallel-accelerated Tool for Genome-wide Association Study. *Genomics Proteomics Bioinformatics* **19**, 619-628 (2021).

15 Jahan, N. *et al.* QTL analysis for rice salinity tolerance and fine mapping of a candidate locus qSL7 for shoot length under salt stress. *Plant Growth Regul.* **90**, 307-319 (2019).

16 Chen, Y. *et al.* Nuclear translocation of OsMFT1 that is impeded by OsFTIP1 promotes drought tolerance in rice. *Mol. Plant* **14**, 1297-1311 (2021).

17 Puram, VRR. *et al.* Identification of QTLs for salt tolerance traits and prebreeding lines with enhanced salt tolerance in an introgression line population of rice. *Plant Mol. Biol. Rep.* **36**, 695–709 (2018).

18 Wang, Z. *et al.* Identification of QTLs with main, epistatic and QTL x environment interaction effects for salt tolerance in rice seedlings under different salinity conditions. *Theor. Appl. Genet.* **125**, 807–815 (2012).

19 Bizimana, JB. *et al.* Identification of quantitative trait loci for salinity tolerance in rice (Oryza sativa L.) using IR29/ Hasawi mapping population. *J. Genet.* **96**, 571–582 (2017).

20 Takehisa, H. *et al.* Identification of quantitative trait loci for plant growth of rice in paddy field flooded with salt water. *Field Crops Res.* **89**, 85–95 (2004).

21 Hossain, H. *et al.* Mapping of quantitative trait loci associated with reproductive stage salt tolerance in rice. *J. Agron. Crop Sci.* **201**, 17–31 (2015).

22 Rahman, MA. *et al.* Mapping QTLs using a novel source of salinity tolerance from Hasawi and their interaction with environments in rice. *Rice* **10**, 47 (2017).

23 De Leon, TB. *et al.* Molecular dissection of seedling salinity tolerance in rice (Oryza sativa L.) using a high-density GBS-based SNP linkage map. *Rice* **9**, 52 (2016).
